# Evaluation of Changes in Prices and Purchases Following Implementation of Sugar-Sweetened Beverage Taxes Across the US

**DOI:** 10.1001/jamahealthforum.2023.4737

**Published:** 2024-01-05

**Authors:** Scott Kaplan, Justin S. White, Kristine A. Madsen, Sanjay Basu, Sofia B. Villas-Boas, Dean Schillinger

**Affiliations:** 1 Department of Economics, US Naval Academy, Annapolis, Maryland; 2 Department of Health Law, Policy & Management, Boston University School of Public Health, Boston, Massachusetts; 3 School of Public Health, University of California, Berkeley; 4 Institute of Health Policy, Management and Evaluation, University of Toronto, Ontario, Canada; 5 Department of Agricultural & Resource Economics, University of California, Berkeley; 6 Division of General Internal Medicine, Center for Vulnerable Populations, San Francisco General Hospital/University of California, San Francisco

## Abstract

**Importance:**

Sugar-sweetened beverage (SSB) taxes are promoted as key policies to reduce cardiometabolic diseases and other conditions, but comprehensive analyses of SSB taxes in the US have been difficult because of the absence of sufficiently large data samples and methods limitations.

**Objective:**

To estimate changes in SSB prices and purchases following SSB taxes in 5 large US cities.

**Design, Setting, and Participants:**

In this cross-sectional study with an augmented synthetic control analysis, changes in prices and purchases of SSBs were estimated following SSB tax implementation in Boulder, Colorado; Philadelphia, Pennsylvania; Oakland, California; Seattle, Washington; and San Francisco, California. Changes in SSB prices (in US dollars) and purchases (volume in ounces) in these cities in the 2 years following tax implementation were estimated and compared with control groups constructed from other cities. Changes in adjacent, untaxed areas were assessed to detect any increase in cross-border purchases. Data used for this analysis spanned from January 1, 2012, to February 29, 2020, and were analyzed between June 1, 2022, and September 29, 2023.

**Main Outcomes and Measures:**

The main outcomes were the changes in SSB prices and volume purchased.

**Results:**

Using nutritional information, 5500 unique universal product codes were classified as SSBs, according to tax designations. The sample included 26 338 stores—496 located in treated localities, 1340 in bordering localities, and 24 502 in the donor pool. Prices of SSBs increased by an average of 33.1% (95% CI, 14.0% to 52.2%; *P* < .001) during the 2 years following tax implementation, corresponding to an average price increase of 1.3¢ per oz and a 92% tax pass-through rate from distributors to consumers. SSB purchases declined in total volume by an average of 33.0% (95% CI, −2.2% to −63.8%; *P* = .04) following tax implementation, corresponding to a −1.00 price elasticity of demand. The observed price increase and corresponding volume decrease immediately followed tax implementation, and both outcomes were sustained in the months thereafter. No evidence of increased cross-border purchases following tax implementation was found.

**Conclusions and Relevance:**

In this cross-sectional study, SSB taxes led to substantial, consistent declines in SSB purchases across 5 taxed cities following price increases associated with those taxes. Scaling SSB taxes nationally could yield substantial public health benefits.

## Key Points

**Question:**

What changes occurred in sugar-sweetened beverage (SSB) prices and purchase volume after SSB taxes were implemented in 5 large US cities?

**Findings:**

In this cross-sectional study, SSB taxes in Boulder, Colorado; Philadelphia, Pennsylvania; Oakland, California; San Francisco, California; and Seattle, Washington, were associated with a 33.1% composite increase in SSB prices (92% pass-through of taxes to consumers) and a 33% reduction in purchase volume, without increasing cross-border purchases. The results were sustained in the months following tax implementation.

**Meaning:**

The results suggest substantial, consistent declines in SSB purchases across several US cities; insofar as reducing SSB consumption can improve population health, scaling SSB taxes more broadly should be considered.

## Teaser

This cross-sectional study evaluates the changes in prices and purchase volume of sugar-sweetened beverages (SSBs) in the 2 years following implementation of SSB taxes in 5 US cities.

## Introduction

Sugar-sweetened beverages (SSBs) are a major source of nonnutritional calories and are associated with serious adverse health outcomes, including type 2 diabetes, obesity, cardiovascular disease, gum disease, caries, and others that contribute to morbidity and mortality.^1,2^ Because of the associations between SSBs and these outcomes, excise taxes on SSBs have been proposed in the US and around the world. As of November 2022, 8 US jurisdictions and more than 50 countries have implemented some form of SSB tax.^3^ Several systematic reviews and meta-analyses have examined the association of SSB excise taxes with both prices and consumption.^4,5,6^ The most recent international review finds a pass-through rate from distributors to consumers of 82% (95% CI, 66%-98%), a mean reduction in SSB sales of 15% (95% CI, −20% to −9%), and an average demand elasticity of −1.59 (95% CI, −2.11 to −1.08).^7^

Yet, nearly all US-based studies of SSB taxes analyzed 1 taxed city and compared it with a control city. To our knowledge, only 2 existing studies have evaluated joint estimates of SSB taxes across multiple taxed cities.^8,9^ However, recent statistical advances suggest that these estimates likely suffer from bias associated with conventional 2-way fixed effects (TWFE) approaches that cannot account for time-varying confounders, which differ between experimental and control populations.^10^ Unbiased estimation of a composite effect, which provides a pooled estimate of SSB taxes across multiple taxed cities, is critical for understanding the generalizability of SSB tax outcomes to different localities with heterogeneous characteristics; such an estimate is complementary to existing estimates from individual localities with SSB taxes in place. This estimate, though imperfect, also better informs the potential effectiveness of a nationwide tax, which was recommended by a recent federal commission on diabetes^11^ and is especially relevant considering the beverage industry’s recent efforts to preempt localities from levying SSB taxes.^12^

In this cross-sectional study with an augmented synthetic control (ASC) analysis, retail sales data from Boulder, Colorado; Philadelphia, Pennsylvania; Oakland, California; Seattle, Washington; and San Francisco, California, were used to estimate the composite effect of SSB taxes on SSB prices and volume purchased. We applied recent advances in statistical methods to estimate an ASC model with staggered adoption, which produces joint estimates from taxes in several treated cities, despite different timing of policy implementation. Unlike conventional TWFE approaches, an ASC model with staggered adoption addresses time-invariant and time-varying unobserved confounders that differ between taxed cities and their untaxed comparators.^10,13,14^ We also estimated composite changes in cross-border shopping in untaxed adjacent areas to examine if consumers offset SSB purchases in bordering localities following SSB tax implementation.

## Methods

This study followed the Strengthening the Reporting of Observational Studies in Epidemiology (STROBE) reporting guideline for cross-sectional studies.^15^ Informed consent was waived because the data were deidentified. The research was determined not to meet the criteria for human participant research by the institutional review board at the University of California, San Francisco. The data used in this analysis spanned from January 1, 2012, to February 29, 2020, and were analyzed between June 1, 2022, and September 29, 2023.

Retail scanner data on SSB prices (in US dollars) and volume sold (in ounces) and a staggered adoption ASC approach were used to estimate the composite change in prices and purchases following the implementation of SSB taxes in Boulder, Philadelphia, Oakland, Seattle, and San Francisco. We also estimated composite changes in cross-border shopping using adjacent, untaxed areas.

### Data Collection

The primary data set was the Nielsen Corporation’s retail scanner data. It consisted of product-week-store observations from selected chain stores in nearly all 3-digit zip codes across the US (871) over the study period from January 1, 2012, to February 29, 2020. The data included total units sold and the average sale price per unit for each observation. Beverage products from this data set were supplemented with nutritional and general product information from Label Insight (Nielsen Consumer LLC)^16^ and hand-coded nutritional information. This enabled the classification of individual beverage products as SSBs or not, on the basis of tax regulations across the 5 cities. Artificially sweetened beverages were not included in the analysis, despite coverage in Philadelphia’s SSB tax. The eMethods in Supplement 1 contain additional details on product selection and tax status classification procedures.

The Table provides a summary of information about the study’s localities. There were 5 taxed 3-digit zip codes examined: 803 (Boulder), 191 (Philadelphia), 946 (Oakland), 981 (Seattle), and 941 (San Francisco). Each of these 3-digit zip codes formed the full set of taxed jurisdictions. The California cities Berkeley and Albany (947) were not included because they were taxed at different times and could not be separately identified from one another (see Limitations). Localities with sales taxes, which include the District of Columbia and Navajo Nation, were omitted because they tend to be smaller in magnitude and less likely to change purchasing behavior. Among the 5 treated localities in this cross-sectional study, the 3 dates in which SSB taxes were implemented varied by city—in Philadelphia, the SSB tax was implemented January 1, 2017; in Boulder and Oakland, July 1, 2017; and in San Francisco and Seattle, January 1, 2018. Tax amounts ranged from 1¢ per oz to 2¢ per oz. Cross-border purchasing was examined in all immediately adjacent 3-digit zip codes, of which there were 13 (Table). These areas did not contain any taxed jurisdictions.

**Table.** Descriptive Statistics of 3-Digit Zip Codes in Primary Analysis

Variable	Three-digit zip code						
	941 (SF)	946 (Oak)	191 (Phil)	803 (Boul)	981 (Sea)	Bordering localities^a^	Donor pool^b^
Three-digit zip codes, No.	1	1	1	1	1	13	279
Stores, No.	103	41	213	26	113	1340	24 502
Date tax implemented	January 1, 2018	July 1, 2017	January 1, 2017	July 1, 2017	January 1, 2018	NA	NA
Pretax months in data, No.	72	66	60	66	72	NA	NA
Posttax months in data, No.	24	30	36	30	24	NA	NA
Dollars per oz	0.01	0.01	0.015	0.02	0.0175	NA	NA

Abbreviations: Boul, Boulder, Colorado; NA, not applicable; Oak, Oakland, California; Phil, Philadelphia, Pennsylvania; Sea, Seattle, Washington; SF, San Francisco, California.

^a^ Bordering 3-digit zip codes comprise all immediately adjacent 3-digit zip codes to each of the 5 treated zip codes, including 800, 804, 805, 945, 948, 080, 081, 940, 949, 980, 982, 983, and 984.

^b^ Donor zip codes consist of all 3-digit zip codes with a % urban value within 1 SD (0.35) of the mean urbanicity of the 5 treated localities (0.98).

### Outcome Variables

Two primary outcome measures were examined, including the monthly change in total number of ounces of SSB products sold in treated localities compared with the synthetic control localities following tax implementation. Total ounces of SSB products sold was the outcome used in the cross-border shopping analysis.

### Statistical Analysis

This cross-sectional study used an ASC approach.^17^ The original synthetic control method uses a data-driven approach to construct a synthetic control unit as a weighted average of all potential control units that best match the treated unit on both the pretreatment outcome and prognostic factors.^18^ The ASC approach extends this method by (1) allowing for multiple treated units experiencing treatment at different times and (2) providing a robust correction procedure when the synthetic unit’s pretreatment outcomes do not closely match those of the treated units. Using a donor pool of untaxed, nonbordering 3-digit zip codes, a synthetic treated unit was constructed for each of the 5 treated cities using pretax SSB prices and purchases, as well as a set of time-invariant characteristics from the 2010 Decennial Census and 2016 American Community Survey.^19,20^ Data were analyzed using R statistical software, version 4.3.2 (R Project for Statistical Computing).

#### Price Pass-Through and Volume Sold

The primary ASC analyses were estimated at the 3-digit zip code–by-month level. We used the weighted average shelf price of SSBs and aggregated the total ounces purchased of SSBs at this unit of observation. Then, separate estimations assessed the composite posttax implementation change in shelf prices and volume sold in treated localities compared with a synthetic locality for each. Each individual city was given equal weight in calculating the composite outcome. The percentage change in shelf prices and volume sold was computed using pretax average shelf prices and volume sold in the treated localities.

**Figure 1.**
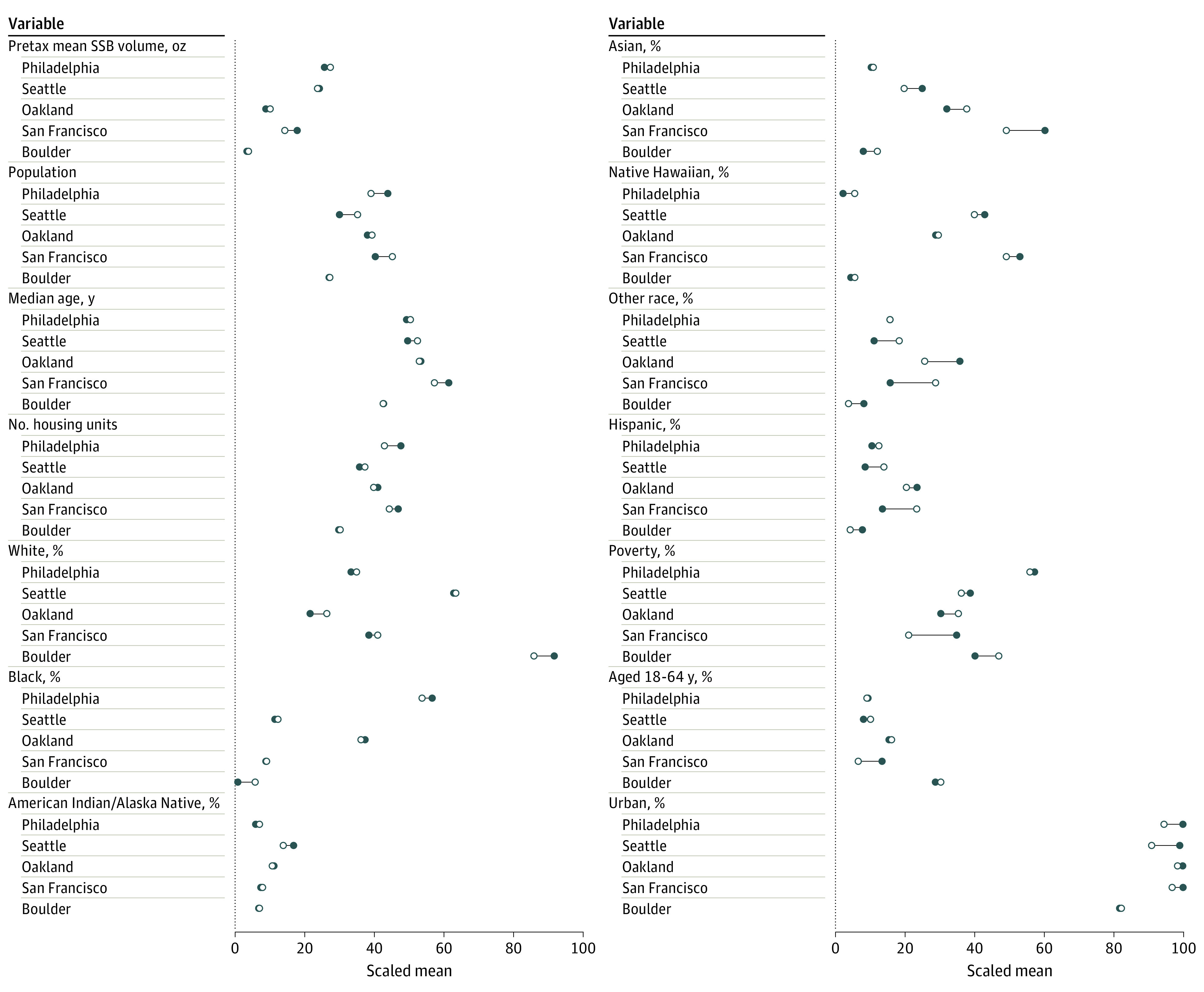
Treated vs Synthetic Values of Pretreatment Sugar-Sweetened Beverage (SSB) Sales Volume and Sociodemographic Characteristics This plot shows the scaled mean values of pretax outcomes and prognostic covariates included in the synthetic control analysis of SSB volume purchased. Mean values are scaled to be between 0 and 100 on the basis of each variable’s maximum and minimum values found in the primary sample. Shaded dots correspond to the mean value for a treated city, and hollow dots correspond to its synthetic control. Other race and ethnicity, as determined by the 2010 US Census, included multiracial and Hispanic individuals.

Adhering to the approach in the study by Abadie,^21^ the donor pool was limited to units with similar characteristics, namely jurisdictions within 1 SD (0.35) of the mean urbanicity level of the 5 treated localities (0.98), following the US Census definition of urban vs rural. A total of 284 three-digit zip codes remained, including the 5 treated localities, but omitting the 13 border localities. Sociodemographic and geographic characteristics used in constructing the synthetic units are shown in Figure 1 and described in the second section of the eMethods in Supplement 1. These characteristics were chosen on the basis of previous research examining SSB taxes.^22,23,24,25^

To determine the statistical significance of the ASC average treatment effects, which are calculated as the average posttax percentage changes in SSB prices and purchases for treated units relative to that of the synthetic control units, placebo estimates were generated for each donor unit one by one, as if each of those units had been treated.^18^ Because treated localities implemented taxes at different times, this procedure was repeated for each treated locality, generating 279 × 5 = 1395 placebo estimates. To generate *P* values, the ratio of mean squared prediction error in the posttax vs pretax period was computed for the composite unit estimate and each placebo estimate, which were then ranked from largest to smallest.^26^ The *P* value was calculated as the ratio of the composite unit ranking to the total number of units (1396) and indicated statistical significance when *P* < .05. More details are provided in the eMethods in Supplement 1.

#### Cross-Border Purchasing

To fully quantify the changes following SSB taxes in treated cities, we also explored whether purchasing behavior changed in adjacent 3-digit border zip codes. The same ASC procedure was implemented, except all adjacent border localities were considered treated, and taxed cities were excluded. Because border localities tended to be semiurban or suburban, the subsample of donor pool units was modified to those featuring an urbanicity level within 1 SD (0.35) of the mean urbanicity of the 13 border localities (0.75). A total of 369 three-digit zip codes remained, including the 13 border localities. This analysis used the same Census characteristics and *P* value calculation approach.

#### Sensitivity Analysis

To assess sensitivity, 2 different urbanicity cutoffs were used to determine the donor pool subsample. Both an urbanicity level of 0.9 and 0.85 were used, reducing the donor pool of 3-digit zip codes to 204 and 226, respectively.

## Results

### Sample Composition

The main analytic sample included 28 512 three-digit zip code–by-month observations from 297 three-digit zip codes across 98 months. Using nutritional information from the supplementary hand-coded and Label Insight data, 5500 unique universal product codes (UPCs) were confirmed as SSBs according to the tax designations. The sample included 26 338 stores—496 located in treated localities, 1340 in bordering localities, and 24 502 in the donor pool. The Table provides summary information for each group of localities.

Figure 1 compares each treated unit with its corresponding synthetic unit, focusing on pretax mean SSB volume in ounces and the 12 sociodemographic and geographic covariates. (In Supplement 1, eFigure 1 displays the price analysis comparisons.) Variables were scaled to be between 0 and 100, so that the units of measure were comparable. In most instances, these values were highly similar (within 5 index points), and no comparisons differed by more than 14 index points. In Supplement 1, eFigure 2 displays sample distributions of each Census characteristic.

### ASC Analyses of SSB Prices and Volume Sold

**Figure 2.**
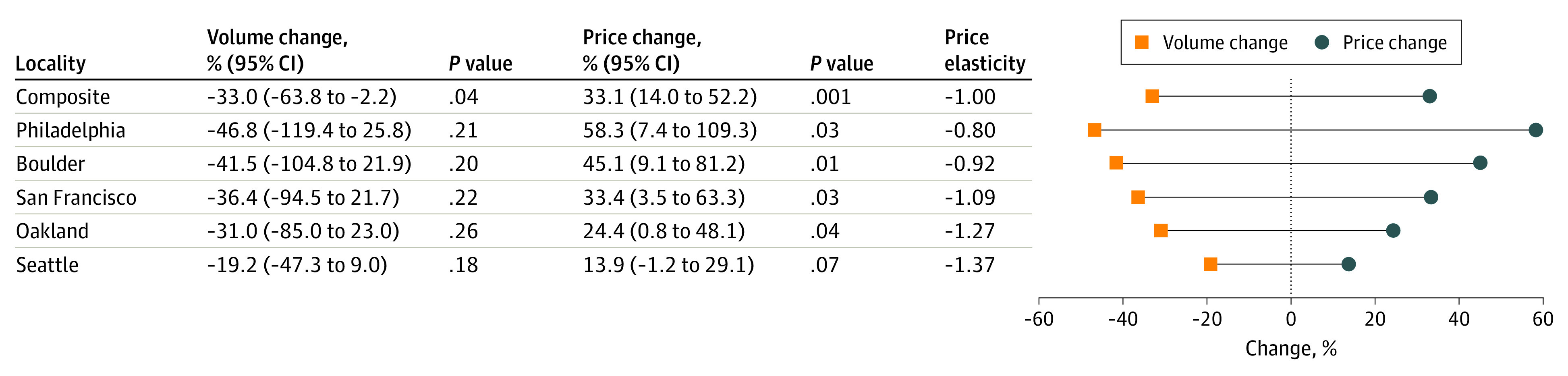
Composite and Individual Locality Demand Elasticity Estimates This plot shows the percentage change in volume sold measured in ounces (orange squares) and the percentage change in shelf prices measured in US dollars (blue circles) for the augmented synthetic control with staggered adoption composite analysis. The plot shows the same information for the augmented synthetic control analyses of the 5 treated localities individually. Price elasticities of demand are provided, and 95% CIs and *P* values for each percentage change in price or volume are also provided.

In the composite treated locality, shelf prices of SSB products increased by an average of 33.1% (95% CI, 14.0%-52.2%; *P* < .001) in the 2 years following tax implementation, relative to the average percentage change in the composite synthetic locality. This corresponded to an average price increase of 1.3¢ per oz (Figure 2) and a 92% price pass-through rate (eFigure 3 in Supplement 1). The volume of SSBs purchased declined by an average of 33.0% (95% CI, −2.2% to −63.8%; *P* = .04) during the same time frame, relative to the average percentage change in the composite synthetic locality. This corresponded to an average monthly change of 18 534 oz/store-month (Figure 2). Together, these estimates yielded a −1.00 price elasticity of demand, suggesting SSB purchasing behavior was responsive to changes in shelf prices (Figure 2). Figure 2 also shows changes in shelf prices and volume purchased for the 5 taxed localities individually. The demand elasticity estimates were relatively consistent across taxed localities, ranging from −0.80 (Philadelphia) to −1.37 (Seattle). Shelf price changes for individual cities were significant at the 10% level, yet null changes in volume purchased could not be rejected for each city at the 10% level.

**Figure 3.**
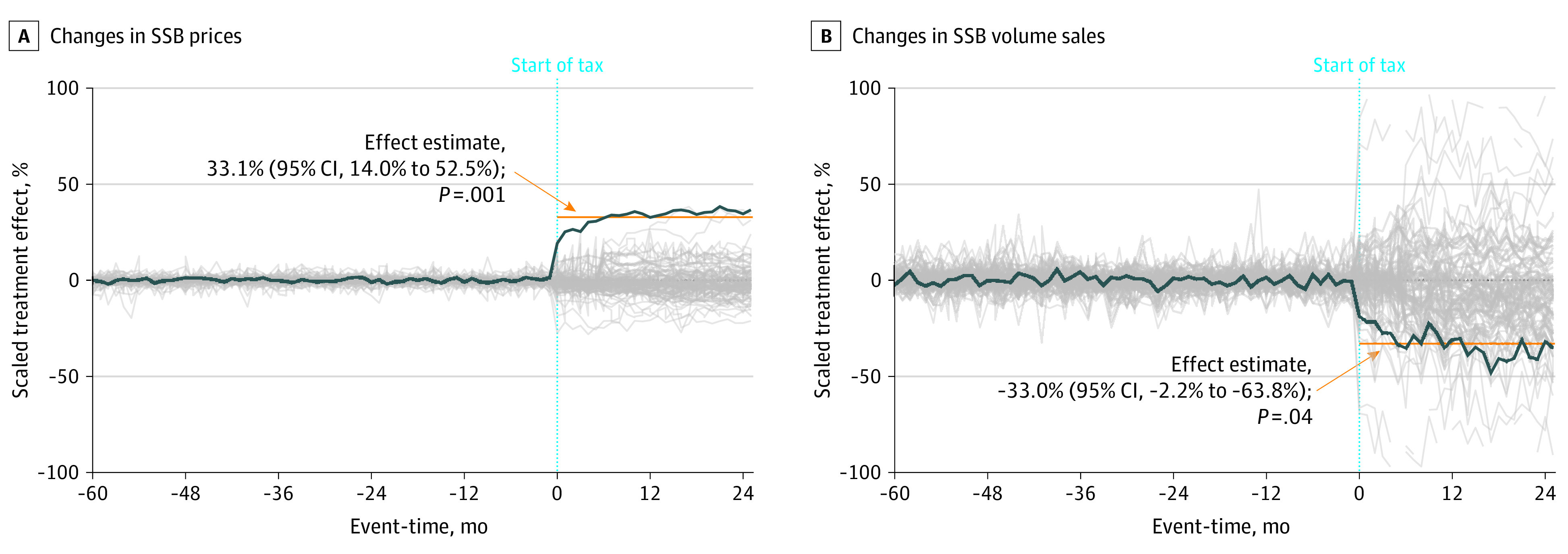
Augmented Synthetic Control Estimates for Composite Changes in Sugar-Sweetened Beverage (SSB) Price and Sales Volume A, This panel shows the percentage change in shelf prices (in US dollars) in response to implementing an excise SSB tax for the staggered adoption composite analysis. B, This panel shows the percentage change in volume sold (in oz). The blue line represents the composite treated unit, and the gray lines represent in-space placebo estimates from the donor pool, which comprise untaxed localities. Percentage changes are calculated for the average of the pretreatment means of each of the 5 treated localities. The light blue dotted line represents the start of the SSB tax. The composite effect size estimates and *P* values are provided in each panel.

Figure 3A shows time-varying ASC results for SSB shelf prices, and Figure 3B shows this information for volume sales. The blue line indicates the difference between the composite treated unit and synthetic unit, and the gray lines represent each placebo estimate. In both analyses, a close fit between the composite treated unit and synthetic unit was found in the pretax period. There was a steep, immediate increase in shelf prices and decrease in volume sales following tax implementation, which was sustained in the months thereafter.

Each city in the composite analysis was equally weighted. The procedure and context through which each city introduced an SSB tax varies, and the findings are intended for policymakers considering tax implementation in specific geographies. The population-weighted composite estimates are similar (eFigures 8 and 9 in Supplement 1).

The analyses for different urbanicity cutoffs generated similar results (eFigures 10 and 13 in Supplement 1). In Supplement 1, eFigures 5 and 6 show the individual city ASC analyses.

### ASC Analyses of Cross-Border Shopping

**Figure 4.**
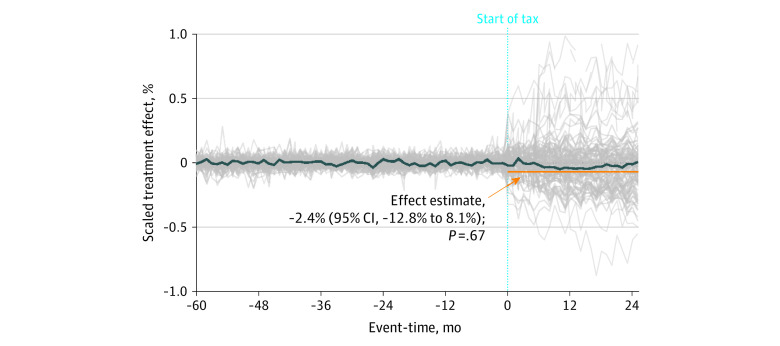
Augmented Synthetic Control Estimates of Composite Changes in Sugar-Sweetened Beverage (SSB) Sales Volume in Border Areas This figure shows the percentage change in volume sold (in oz) from the staggered adoption composite analysis in immediately adjacent bordering 3-digit zip codes. These data were examined in response to implementing an excise SSB tax in the 5 treated zip codes. The dark blue line represents the composite adjacent border unit, and the gray lines represent in-space placebo estimates from the donor pool. Percentage changes are calculated for the average of the pretreatment means of each of the 12 adjacent border localities. The light blue dotted line represents the start of the SSB tax. The composite effect size estimates and *P* values are provided.

Figure 4 shows the time-varying ASC results for cross-border SSB volume sales. There was no statistically significant mean change in cross-border purchases of SSBs following tax implementation (−2.4%; 95% CI, −12.8% to 8.1%; *P* = .67), which remained stable in the years following the tax. No significant change in cross-border SSB volume purchases was observed in each taxed city (eFigure 4 in Supplement 1). Estimates for different urbanicity cutoffs provided similar findings (eFigures 12 and 15 in Supplement 1). In Supplement 1, eFigure 7 displays the time-varying cross-border analyses for each taxed city.

## Discussion

In this cross-sectional study with an ASC analysis, SSB excise taxes were associated with large, consistent declines in SSB purchases across 5 US taxed cities following tax-driven price changes. Quasi-experimental methods were used to estimate the overall changes following SSB taxes implemented at different times and locations relative to a synthetic control of untaxed areas. The results show shelf prices of SSB products increased by an average of 33.1% (1.3¢ per oz) in the years following SSB tax implementation, corresponding to a 92% price pass-through rate from distributors to consumers. Volume sales fell by 33.0% during the same time frame, without evidence of changes in cross-border shopping in untaxed adjacent areas.

Although the estimates generally support previous estimates from single-city studies, these results help answer the critical question of how much variation across taxed localities is due to the unique characteristics of a locality vs the generalizable outcomes of a tax. Compared with a recent international meta-analysis of SSB taxes, the results suggest a slightly higher pass-through rate, a substantially larger reduction in volume purchased, and moderately less demand responsiveness to price changes.^6^ These modest discrepancies may reflect differences in geographic areas of comparators, store sample composition, and greater accounting of unmeasured confounders in this analysis than in previous studies. Additionally, conflicting findings have been found regarding cross-border purchasing following SSB taxes, with some studies pointing to significant increases and others finding no changes.^27,28,29,30^ The results provided no evidence of changes in cross-border purchasing.

To further contextualize the findings, we estimated a TWFE event-study model, detailed in the third section of the eMethods in Supplement 1. This model has been the primary approach taken in previous SSB tax evaluation studies. In Supplement 1, eTable 3 shows the point estimates are generally comparable with the ASC estimates, although some moderate differences exist. Inspection of the prepolicy coefficients in the event-study plots suggests that these estimates have varying degrees of bias associated with imperfect pretrends (eFigures 16-19 in Supplement 1).^31,32^ The TWFE estimates are much more precisely estimated than the ASC estimates, in part because the TWFE CIs may be overly narrow.^33,34,35^ Nevertheless, this trade-off highlights this study’s focus on generating unbiased estimates at the partial expense of precision.

It is important to interpret these estimates in the context of projected health benefits. Several studies have found that a 15% to 20% increase in price/decrease in consumption generates significant health benefits, including reductions in myocardial infarction events, ischemic heart disease, coronary heart events, strokes, diabetes, and obesity.^36,37,38^ This study estimated a 33.1% increase in price and a corresponding 33.0% decrease in volume, suggesting health benefits at least as substantial as those found previously.

Additionally, studies have suggested that SSB taxes are highly cost-effective.^22,37,39^ Wang et al^37^ found a nationwide tax could have avoided $17 billion in medical costs between 2010 and 2020. Lee et al^39^ found approximately $53 billion in cost savings throughout an average individual lifetime. More recently, White et al^22^ found that a 27% reduction in consumption in Oakland is expected to accrue more than $100 000 per 10 000 residents in societal cost savings during a 10-year period. This study’s findings suggest SSB taxation would likely generate significant improvements in population health and substantial cost savings.

### Limitations

First, the retail scanner data identify purchasing behavior and not direct consumption. It is possible, though unlikely, that taxed populations consumed a different share of purchased SSBs than did untaxed control populations (eg, producing more waste). Second, the data were geocoded by 3-digit zip code. This prevented Berkeley and Albany (3-digit zip code 947) from being included because they could not be separately identified and were taxed at different times. The 3-digit zip codes for included taxed cities contained a small number of untaxed jurisdictions, accounting for less than 7% of the total population of these areas (eTable 2 in Supplement 1). However, this misclassification should only lead to an underestimate of the changes following tax implementation.

We also lacked nutritional information for certain beverage UPCs. Of the UPCs of SSBs in the scanner data, we successfully matched 84.0% of sales volume in ounces using Label Insight and hand-coded data featuring nutritional information. To the extent that the set of unmatched UPCs was similar across taxed and untaxed jurisdictions, the findings should be unaffected. Additionally, the scanner data contained only a subsample of all stores in each zip code; thus, the data did not include all volume sales. Using SSB tax revenues to estimate total volume sales in treated localities, coverage from this set of products was 12.7% (eTable 1 in Supplement 1). The coverage estimates were similar but slightly lower than recent SSB tax evaluations using Nielsen data.^29,40^ Lower coverage in Philadelphia was partially due to the exclusion of artificially sweetened beverages from this analysis. Coverage could not be calculated in donor zip codes because there were no SSB taxes in place. However, the ASC estimation generated a reliable counterfactual group from the existing sample of donor zip codes, which should mitigate any unintended bias caused by unequal SSB coverage across treatment and control localities.

Next, although the ASC estimates for each individual city in the volume analysis (Figure 2; eFigure 6 in Supplement 1) were similar to those in prior studies,^7^ they were relatively imprecise, and a null effect could not be rejected at the 5% level. Furthermore, although the composite estimates for the volume analysis were much more precise, reductions in purchases as small as 2% or as large as 64% could not be ruled out at a 95% CI level. While synthetic control methods deliver less biased estimates than difference-in-differences approaches, they also generate less statistical power.^41^ However, difference-in-differences studies involving a small number of treated units may underestimate the true variance of effect estimates.^33,34,35^ As more localities introduce SSB taxes, synthetic control methods with staggered adoption will have greater precision.

In addition, only posted shelf prices were observed in the scanner data, which may lead to underestimates of pass-through rates. While excise taxes are generally reflected in shelf prices, certain retailers may have only included the tax once products were scanned at the register.^42^ Moreover, the scanner data were primarily composed of information from large chain stores. Thus, these results may not extend to independent stores, although similar estimates have been found in those settings.^43^ Finally, the 5 treated localities studied here, while geographically distinct and racially, ethnically, and socioeconomically diverse, were not fully representative of the US population. Therefore, the findings may not be fully generalizable on a national scale, a limitation most relevant to less urban populations.

## Conclusions

In this cross-sectional study with an ASC analysis, SSB taxes in Boulder, Philadelphia, Oakland, San Francisco, and Seattle were associated with 33.1% composite increases in SSB prices (92% pass-through rate) and 33.0% reductions in SSB purchases, with no offset through cross-border purchases of SSBs. The changes in prices and purchases remained stable in the years following tax implementation. The findings have important implications for the potential efficacy of SSB taxes across larger geographic jurisdictions and at the national level. Scaling SSB excise taxes across the US would likely generate significant population health benefits and medical cost savings.

## Notes

Supplement 1. **eMethods.** Supplemental Methods **eTable 1.** Total Coverage of SSB Ounces Sold in Matched Nielsen Retail Scanner Data **eTable 2.** Total Population (2010) by City within Taxed 3-Digit Zip Codes **eTable 3.** Two-Way Fixed Effects Estimation Results for Composite and Individual City Analyses **eFigure 1.** Comparing Treated and Synthetic Values of Prognostic Factors from the Analysis of SSB Shelf Prices **eFigure 2.** Overlap of US Census Sociodemographic Characteristics Between Each Taxed City and the Donor Pool of Control 3-Digit Zip Codes **eFigure 3.** Composite and Individual Locality Price Pass-Through **eFigure 4.** Composite and Individual Changes in Volume Sales in Adjacent Border Zip Codes **eFigure 5.** Augmented Synthetic Control Estimates for Individual Locality Changes in Price **eFigure 6.** Augmented Synthetic Control Estimates for Individual Locality Changes in Volume Sales **eFigure 7.** Augmented Synthetic Control Estimates of Individual Locality Changes in Volume Sales of SSB Products in Border Areas **eFigure 8.** Augmented Synthetic Control Estimates for Composite Changes in Price and Volume Sales of SSB Products (Population Weighted) **eFigure 9.** Augmented Synthetic Control Estimates of Composite Changes in Volume Sales of SSB Products in Border Areas (Population Weighted) **eFigure 10.** Composite and Individual Locality Demand Elasticity Estimates (Urbanicity > 0.85) **eFigure 11.** Augmented Synthetic Control Estimates for Composite Changes in Price and Volume Sales of SSB Products (Urbanicity > 0.85) **eFigure 12.** Augmented Synthetic Control Estimates of Composite Changes in Volume Sales of SSB Products in Border Areas (Urbanicity > 0.85) **eFigure 13.** Composite and Individual Locality Demand Elasticity Estimates (Urbanicity > 0.9) **eFigure 14.** Augmented Synthetic Control Estimates for Composite Changes in Price and Volume Sales of SSB Products (Urbanicity > 0.9) **eFigure 15.** Augmented Synthetic Control Estimates of Composite Changes in Volume Sales of SSB Products in Border Areas (Urbanicity > 0.9) **eFigure 16.** TWFE Estimates of Composite Changes in Prices, Volume Sales, and Border Volume Sales **eFigure 17.** TWFE Estimates of Individual Locality Changes in Prices **eFigure 18.** TWFE Estimates of Individual Locality Changes in Volume Sales **eFigure 19.** TWFE Estimates of Individual Locality Changes in Volume Sales of SSB Products in Border Areas

Supplement 2.
Data Sharing Statement
