# Error in Figure

**DOI:** 10.1001/jamahealthforum.2024.0017

**Published:** 2024-02-16

The Original Investigation titled “Evaluation of Changes in Prices and Purchases Following Implementation of Sugar-Sweetened Beverage Taxes Across the US,”^1^ published on January 5, 2024, was corrected to fix the upper 95% CI value in Figure 3A from 52.5% to 52.2%. This article was corrected online.
