# Supplementary material for: Evaluation of Changes in Prices and Purchases Following Implementation of Sugar-Sweetened Beverage Taxes Across the US
Source: JAMA Health Forum. 2024 Jan 5;5(1):e234737. doi: 10.1001/jamahealthforum.2023.4737 (PMC10770775; doi:10.1001/jamahealthforum.2023.4737)
Supplement: Supplement 1. — eMethods. Supplemental Methods eTable 1. Total Coverage of SSB Ounces Sold in Matched Nielsen Retail Scanner Data eTable 2. Total Population (2010) by City within Taxed 3-Digit Zip Codes eTable 3. Two-Way Fixed Effects Estimation Results for Composite and Individual City Analyses eFigure 1. Comparing Treated and Synthetic Values of Prognostic Factors from the Analysis of SSB Shelf Prices eFigure 2. Overlap of US Census Sociodemographic Characteristics Between Each Taxed City and the Donor Pool of Control 3-Digit Zip Codes eFigure 3. Composite and Individual Locality Price Pass-Through eFigure 4. Composite and Individual Changes in Volume Sales in Adjacent Border Zip Codes eFigure 5. Augmented Synthetic Control Estimates for Individual Locality Changes in Price eFigure 6. Augmented Synthetic Control Estimates for Individual Locality Changes in Volume Sales eFigure 7. Augmented Synthetic Control Estimates of Individual Locality Changes in Volume Sales of SSB Products in Border Areas eFigure 8. Augmented Synthetic Control Estimates for Composite Changes in Price and Volume Sales of SSB Products (Population Weighted) eFigure 9. Augmented Synthetic Control Estimates of Composite Changes in Volume Sales of SSB Products in Border Areas (Population Weighted) eFigure 10. Composite and Individual Locality Demand Elasticity Estimates (Urbanicity > 0.85) eFigure 11. Augmented Synthetic Control Estimates for Composite Changes in Price and Volume Sales of SSB Products (Urbanicity > 0.85) eFigure 12. Augmented Synthetic Control Estimates of Composite Changes in Volume Sales of SSB Products in Border Areas (Urbanicity > 0.85) eFigure 13. Composite and Individual Locality Demand Elasticity Estimates (Urbanicity > 0.9) eFigure 14. Augmented Synthetic Control Estimates for Composite Changes in Price and Volume Sales of SSB Products (Urbanicity > 0.9) eFigure 15. Augmented Synthetic Control Estimates of Composite Changes in Volume Sales of SSB Products in Border Areas (Urbanicity > 0.9 [file jamahealthforum-e234737-s001.pdf]

# Supplemental Online Content

Kaplan S, White JS, Madsen KA, Basu S, Villas-Boas SB, Schillinger D. Evaluation of changes in prices and purchases following implementation of sugar-sweetened beverage taxes across the US. *JAMA Health Forum.* 2024;5(1):e234737. doi:10.1001/jamahealthforum.2023.4737

**eMethods.** Supplemental Methods: (1) Nielsen Data and Beverage Classification Procedure; (2) Augmented Synthetic Control Model; (3) Two-Way Fixed Effects (TWFE) Model

**eTable 1.** Total Coverage of SSB Ounces Sold in Matched Nielsen Retail Scanner Data

**eTable 2.** Total Population (2010) by City within Taxed 3-Digit Zip Codes

**eTable 3.** Two-Way Fixed Effects Estimation Results for Composite and Individual City Analyses

**eFigure 1.** Comparing Treated and Synthetic Values of Prognostic Factors from the Analysis of SSB Shelf Prices

**eFigure 2.** Overlap of US Census Sociodemographic Characteristics Between Each Taxed City and the Donor Pool of Control 3-Digit Zip Codes

**eFigure 3.** Composite and Individual Locality Price Pass-Through

**eFigure 4.** Composite and Individual Changes in Volume Sales in Adjacent Border Zip Codes

**eFigure 5.** Augmented Synthetic Control Estimates for Individual Locality Changes in Price

**eFigure 6.** Augmented Synthetic Control Estimates for Individual Locality Changes in Volume Sales

**eFigure 7.** Augmented Synthetic Control Estimates of Individual Locality Changes in Volume Sales of SSB Products in Border Areas

**eFigure 8.** Augmented Synthetic Control Estimates for Composite Changes in Price and Volume Sales of SSB Products (Population Weighted)

**eFigure 9.** Augmented Synthetic Control Estimates of Composite Changes in Volume Sales of SSB Products in Border Areas (Population Weighted)

© 2024 Kaplan S et al. *JAMA Health Forum.*

**eFigure 10.** Composite and Individual Locality Demand Elasticity Estimates (Urbanicity > 0.85)

**eFigure 11.** Augmented Synthetic Control Estimates for Composite Changes in Price and Volume Sales of SSB Products (Urbanicity > 0.85)

**eFigure 12.** Augmented Synthetic Control Estimates of Composite Changes in Volume Sales of SSB Products in Border Areas (Urbanicity > 0.85)

**eFigure 13.** Composite and Individual Locality Demand Elasticity Estimates (Urbanicity > 0.9)

**eFigure 14.** Augmented Synthetic Control Estimates for Composite Changes in Price and Volume Sales of SSB Products (Urbanicity > 0.9)

**eFigure 15.** Augmented Synthetic Control Estimates of Composite Changes in Volume Sales of SSB Products in Border Areas (Urbanicity > 0.9)

**eFigure 16.** TWFE Estimates of Composite Changes in Prices, Volume Sales, and Border Volume Sales

**eFigure 17.** TWFE Estimates of Individual Locality Changes in Prices

**eFigure 18.** TWFE Estimates of Individual Locality Changes in Volume Sales

**eFigure 19.** TWFE Estimates of Individual Locality Changes in Volume Sales of SSB Products in Border Areas

This supplemental material has been provided by the authors to give readers additional information about their work.

© 2024 Kaplan S et al. *JAMA Health Forum.*

**eMETHODS**

## 1. Nielsen Data and Beverage Classification Procedure

The Nielsen retail scanner dataset was made available through a subscription to the Kilts Marketing Center at the University of Chicago Booth School of Business. The data is comprised of ten general product categories: HEALTH AND BEAUTY CARE, DRY GROCERY, FROZEN FOODS, DAIRY, DELI, PACKAGED MEAT, FRESH PRODUCE, NON-FOOD GROCERY, ALCOHOLIC BEVERAGES, GENERAL MERCHANDIZE, and UNCLASSIFIED. We query all products falling within the DRY GROCERY category. Within the DRY GROCERY category, we select the following subcategories of products, which form the sample of UPCs we initially work with: JUICE DRINKS – CANNED, BOTTLED; CARBONATED BEVERAGES; and SOFT DRINKS – NON-CARBONATED.

Because the Nielsen data provides limited nutritional information about each product, we leverage 10-digit UPC data from Label Insight (LI) and hand-coded data from a previous study[1] to classify individual products into SSB vs. non-SSB status. These two datasets include information regarding total calories, total sugar, added sugar per serving, serving size, and presence of artificial sweeteners, which allowed us to ascertain the SSB status for each UPC. Because the Nielsen data provides UPCs in EAN-13 format (with the check digit dropped), and LI and the hand-coded data contain information at the 10-digit UPC level, we convert the Nielsen 12-digit UPCs into 10-digit UPCs. We do so by following the procedure laid out in documentation from Label Insights to merge their UPC data with Nielsen UPC data, i.e. we drop the first two digits of Nielsen UPCs and the first and last digits of Label Insights UPCs. Any UPCs that become duplicated due to this procedure are simply aggregated together. We also took this classification approach with the hand-coded data.

We were successful in matching on 18,147 10-digit UPCs, which make up 84.0% of the total sales volume in the Nielsen beverage data over our study period. Of the matched 10-digit UPCs, 5,500 are classified as SSBs, which account for 39.7% of volume sales in the matched data. This forms the set of products we use in our analyses.

© 2024 Kaplan S et al. *JAMA Health Forum.*

## 2. Augmented Synthetic Control Model

Synthetic control models have become widely used in panel data analyses to assess policy changes.[2-3] These models are advantageous because they algorithmically create a counterfactual unit that can be directly compared to a treated unit of interest without worrying about fundamental differences in outcomes or characteristics of the two groups, by construction. Using our setting as an example, the base synthetic control model matches pre-tax outcomes and covariates of taxed and untaxed units by weighting each untaxed unit in such a way that the "synthetic" unit(s) closely match the taxed unit(s) on both the outcome measure of interest and covariate characteristics. In particular, for each outcome $Y_{im}$ for 3-digit zip code $i$ in month $m$, and $X_i$ 3-digit zip code-level covariates (which in our case are time-invariant), the method chooses weights for each untaxed 3-digit zip code $j$ ($w_j$) to minimize the distance $(Y_{im}, X_i) - \sum_j w_j \left(Y_{jm}, X_j\right)$.[4]

Recent work has extended the initial synthetic control approach in various ways.[5-7] There are two notable enhancements of the original synthetic control method that we leverage in this study. First, the original synthetic control framework was designed to estimate the impact of an intervention on a single treated unit. In our setting, we study multiple treated units that experience treatment at different times, referred to as a "staggered adoption" setup.[8] Second, the use of the original synthetic control method was recommended only when the synthetic unit's pretreatment outcomes closely matched the pre-treatment outcomes of the treated unit. Our study takes advantage of recent work that relaxes this requirement by introducing a "bias-correction" procedure. This estimation framework is called the augmented synthetic control (ASC) model, since it augments the original synthetic control approach with an outcome model that is used to determine bias as a result of a relatively poor pretreatment fit between the treated and synthetic units, and then uses the output to remove the bias in the pretreatment period.[9] While there are several different outcome models that can be used to de-bias the synthetic control model, the primary method used is a ridge regression model.[9] A ridge regression model estimates a linear regression of post-treatment outcomes of the control units $(Y_{jm} | m \geq T)$, where $T$ indicates the month of tax implementation, on the centered pre-

© 2024 Kaplan S et al. *JAMA Health Forum.*

treatment outcomes of the control units ($Y_{jm}|m < T$). This modification allows certain donor units to be assigned negative weights (whereas the original synthetic control procedure restricts all weights on donor units to be $\geq 0$), which can improve pretreatment fit. Additional structure and details of this procedure can be found in sections 2-4 of Ben-Michael, Feller, and Rothstein (2021).

Sociodemographic and geographic characteristics used in constructing synthetic units were taken from the 2010 Decennial Census and 2016 American Community Survey. Characteristics included population size (2010), median household income (2016), racial/ethnic composition (proportion non-Hispanic White, non-Hispanic Black, Hispanic, Asian, and American Indian/Alaska Native 2010), proportion in poverty (household income <$10,000K, 2016), proportion of individuals 18 to 64 years old (2010), number of housing units (2010), and percentage of the population defined as urban (2010).

One important implication of the use of synthetic control methods is the importance of a donor pool consisting of units that could plausibly act as reasonable controls for the treated units.[4] Failure to do so can lead to substantial bias in the estimation. Because of this, we decide to limit the donor pool of 3-digit zip codes to those with urbanicity levels that are similar to the treated units. Using a measure of urbanicity is desirable for different reasons. First, it's easily defined by and computed using information and data from the US Census.[10] Second, urbanicity captures several observed and unobserved characteristics that are likely to influence the relative similarity among control and treated units, including characteristics we include like population, median household income, number of housing units, etc., as well as characteristics we do not observe, like housing prices, police presence, and voter party alignment. Finally, our five treated localities have an average urbanicity level of 0.98, which ranges from a minimum of 0.94 (Boulder) to 1 (Philadelphia and San Francisco). This relative similarity between the treated units' urbanicity allows for the construction of a donor pool that could plausibly act as reasonable controls for each of the treated units, while keeping the donor pool the same for each.

© 2024 Kaplan S et al. *JAMA Health Forum.*

In the primary augmented synthetic control estimation, we use a subsample of control (donor) units that fall within one standard deviation of the average urbanicity level of the five treated localities. In the cross-border shopping analyses, we use a subsample of donor units that fall within one standard deviation of the average urbanicity level of the thirteen adjacent border localities. Robustness checks, which are included in eFigures 10-15, include control units with urbanicity levels >0.85 and >0.9. The results from these supplementary estimations are qualitatively unchanged.

Because implementation of the tax happened at different times across the five treated localities (hence the “staggered adoption” nature of the BCSC procedure), calendar time is converted to event-time, which normalizes time = 0 to the month when the tax went into place in each treated locality. Therefore, in event time we observe a different number of total time periods for each taxed locality. Consequently, we provide results from a “balanced” estimation, which only considers event-time periods when all treated localities are present in the sample. This is done to avoid biasing the estimation in favor of taxed localities that are observed in the data during event-time periods when other taxed localities may not be observed.

To determine the statistical significance of our augmented synthetic control average treatment effects, which are calculated as the average post-tax percent change in SSB purchases (shelf prices), we use an in-space placebo generation inference procedure.[3,11] For each of the five treated localities, we generate in-space placebo estimates for each donor pool unit one-by-one as if each unit had been treated. Because treated localities implement taxes at different times, we repeat this procedure for each of the five different treated localities, which generates 279*5=1,395 placebo estimates. To generate p-values, we compute the ratio of mean squared prediction error (RMSPE) in the post-tax vs. pre-tax period for the composite unit estimate and each of the placebo unit estimates, and rank them from largest to smallest.[11] The p-value for the estimation is calculated as the ratio of the composite unit numerical ranking with respect to the total number of units (1,396). Each of the BCSC plots takes 100 quasi-randomly selected

placebo lines from the universe of 1,396 placebos for the composite estimation and 279 for each of the individual city estimations. This selection procedure is quasi-random in the sense that the universe of eligible placebos to be chosen is "pruned" to those that exhibit a pre-period MSPE that is no greater than five times the pre-period MSPE of the treated unit. Confidence intervals were obtained from p-values using the method outlined by Altman.[12]

### 3. Two-Way Fixed Effects (TWFE) Model

Two-way fixed effects (TWFE) models make up one of the most common empirical approaches to identifying the impact of a treatment (e.g. policy intervention) using panel data.[13-14] This approach has also been often used in the SSB tax evaluation literature.[1,16-17]

Using this conventional approach, we estimate a series of TWFE models and TWFE event study models. The simple TWFE model takes the following form:

$$Y_{it} = \beta Tax_{it} + \alpha_i + \delta_t + \epsilon_{it}$$

Where $Y_{it}$ indicates the outcome variable of interest (i.e. volume purchased or shelf prices) in 3-digit zip code $i$ in month-year $t$, $Tax_{it}$ is a binary variable =1 if 3-digit zip code $i$ has an SSB tax in place during month-year $t$ and =0 otherwise, and $\alpha_i$ and $\delta_t$ represent 3-digit zip code and month-year fixed effects, respectively. $\beta$ measures the treatment effect associated with the implementation of an SSB tax on the outcome variable of interest. We estimate such a TWFE model to determine both the composite effect (by including all five treated 3-digit zip codes) as well as individual city effects (separate estimations for each of the five treated 3-digit zip codes). eTable 3 presents the TWFE estimates for each of these specifications.

We also estimate a TWFE event study specification, which estimates individual coefficients for each month-year in event-time, which is normalized to 0 at the month-year when an SSB tax is implemented in 3-digit zip code $i$. Again, we estimate a TWFE event study to determine both the composite effect (by including all five treated 3-digit zip codes) as well as individual city

© 2024 Kaplan S et al. *JAMA Health Forum.*

effects (separate estimations for each of the five treated 3-digit zip codes). The TWFE event-study model takes the following form:

$$Y_{ite} = \sum_{e=-a \setminus \{-1\}}^{b} \beta_e \, Tax_{e,it} + \alpha_i + \delta_t + \epsilon_{ite}$$

Where $e$ represents the month-year in event-time, ranging from $-a$ to $b$. The period prior to implementation of an SSB tax (-1) is omitted. $\beta_e$ is a vector of coefficients indexed by event-time that can be interpreted relative to the omitted event-time period. $Tax_{e,it} = 1$ if 3-digit zip code $i$ has been treated at event-time $e$. eFigure 16 presents the event study results for the composite estimation, while eFigures 17-19 present the event study results for the individual city estimations.

© 2024 Kaplan S et al. *JAMA Health Forum.*

**REFERENCES**


1. White JS, Basu S, Kaplan S, Madsen KA, Villas-Boas SB, Schillinger D. Evaluation of the sugar-sweetened beverage tax in Oakland, United States, 2015–2019: A quasi-experimental and cost-effectiveness study. PLoS medicine. 2023 Apr 18;20(4):e1004212.
2. Abadie A, Gardeazabal J. The economic costs of conflict: A case study of the Basque Country. American economic review. 2003 Mar 1;93(1):113-32.
3. Abadie A, Diamond A, Hainmueller J. Synthetic control methods for comparative case studies: Estimating the effect of California's tobacco control program. Journal of the American Statistical Association. 2010;105(490):493-505.
4. Abadie A. Using Synthetic Controls: Feasibility, Data Requirements, and Methodological Aspects. J Econ Lit. 2021;59(2):391-425.
5. Abadie A, L'hour J. A penalized synthetic control estimator for disaggregated data. Journal of the American Statistical Association. 2021 Oct 2;116(536):1817-34.
6. Athey S, Bayati M, Doudchenko N, Imbens G, Khosravi K. Matrix Completion Methods for Causal Panel Data Models. arXiv e-prints, page. arXiv preprint arXiv:1710.10251. 2017.
7. Arkhangelsky D, Athey S, Hirshberg DA, Imbens GW, Wager S. Synthetic difference-in-differences. American Economic Review. 2021 Dec 1;111(12):4088-118.
8. Ben-Michael E, Feller A, Rothstein J. Synthetic controls with staggered adoption. Journal of the Royal Statistical Society Series B: Statistical Methodology. 2022 Apr;84(2):351-81.
9. Ben-Michael, Eli, Avi Feller, and Jesse Rothstein. "The augmented synthetic control method." *Journal of the American Statistical Association* 116, no. 536 (2021): 1789-1803.
10. Urban and Rural. US Census. https://www.census.gov/programs-surveys/geography/guidance/geo-areas/urban-rural.html
11. Wiltshire, Justin C. "allsynth: Synthetic Control Bias-Correction Utilities for Stata." In *In2021 Stata Conference*, no. 15. 2021.
12. Altman DG, Bland JM. How to obtain the confidence interval from a P value. Bmj. 2011 Aug 8;343.
13. De Chaisemartin C, d'Haultfoeuille X. Two-way fixed effects estimators with heterogeneous treatment effects. American Economic Review. 2020 Sep 1;110(9):2964-96.
14. Roth J. Pretest with caution: Event-study estimates after testing for parallel trends. American Economic Review: Insights. 2022 Sep 1;4(3):305-22.
15. Cawley, J., Frisvold, D., & Jones, D. (2020). The impact of sugar-sweetened beverage taxes on purchases: Evidence from four city-level taxes in the United States. Health Economics, 29(10), 1289–1306. https://doi.org/10.1002/hec.4141
16. Taylor RL, Kaplan S, Villas-Boas SB, Jung K. Soda wars: The effect of a soda tax election on university beverage sales. Economic Inquiry. 2019 Jul;57(3):1480-96.


© 2024 Kaplan S et al. *JAMA Health Forum.*

**eTable 1.** Total Coverage of SSB Ounces Sold in Matched Nielsen Retail Scanner Data

| City (first complete fiscal year of SSB tax) | Tax Revenue ($000's) | Tax ($/Ounce) | Total SSB Sales (1000s of Ounces) | SSB Sales of Nielsen UPCs (1000s of Ounces) | Coverage (%) |
|---|---|---|---|---|---|
| Boulder (2018) | $4,868 | $.02 | 243,400 | 50,781 | 20.86% |
| Oakland (Jul 2017–Jun 2018) | $11,076 | $.01 | 1,107,600 | 171,850 | 15.52% |
| Philadelphia (Jul 2017–Jun 2018) | $77,421 | $.015 | 5,161,400 | 240,146 | 4.65% |
| San Francisco (Jul 2018–Jun 2019) | $16,098 | $.01 | 1,609,800 | 287,089 | 17.83% |
| Seattle (2018) | $22,254 | $.0175 | 1,271,657 | 404,600 | 31.82% |
| Composite | $131,717 | $.0145 | 9,083,931 | 1,154,468 | 12.71% |

*Note*: Tax revenues taken from Krieger et al. (2021).[1] Coverage estimates use the first fiscal year of each city's respective tax implementation. Lower coverage in Philadelphia is in part due to the exclusion of artificially sweetened beverages in our analysis. The tax amount for the Composite geographic unit is the unweighted average of the tax amounts across the five taxed cities.

[1] Krieger J, Magee K, Hennings T, Schoof J, Madsen KA. How sugar-sweetened beverage tax revenues are being used in the United States. Preventive Medicine Reports. 2021 Sep 1;23:101388.

© 2024 Kaplan S et al. *JAMA Health Forum.*

**eTable 2.** Total Population (2010) by City within Taxed 3-Digit Zip Codes

| 3-Digit Zip Code | City | Tax Status | Population (2010) | % of 3-Digit Zip Code Population | % of Overall Population |
|---|---|---|---|---|---|
| 803 | Boulder | Tax | 97,724 | 100.00% | 2.66% |
| 946 | Oakland | Tax | 391,350 | 94.95% | 10.64% |
| | Emeryville | No Tax | 10,110 | 2.45% | 0.27% |
| | Piedmont | No Tax | 10,709 | 2.60% | 0.29% |
| 191 | Philadelphia | Tax | 1,528,000 | 99.61% | 41.53% |
| | Manayunk | No Tax | 5,913 | 0.39% | 0.16% |
| 941 | San Francisco | Tax | 805,519 | 100.00% | 21.89% |
| 981 | Seattle | Tax | 610,654 | 73.57% | 16.60% |
| | Tukwila | No Tax | 19,161 | 2.31% | 0.52% |
| | Bainbridge Island | No Tax | 23,062 | 2.78% | 0.63% |
| | Shoreline | No Tax | 53,182 | 6.41% | 1.45% |
| | Burien | No Tax | 48,224 | 5.81% | 1.31% |
| | Des Moines | No Tax | 29,775 | 3.59% | 0.81% |
| | Normandy Park | No Tax | 6,335 | 0.76% | 0.17% |
| | Seatac | No Tax | 26,999 | 3.25% | 0.73% |
| | Lake Forest Park | No Tax | 12,639 | 1.52% | 0.34% |
| | TOTAL | Tax | 3,433,247 | -- | **93.31%** |
| | TOTAL | No Tax | 246,109 | -- | **6.69%** |

*Note*: Population estimates for each city taken from 2010 (source: US Census Bureau). In the "981" 3-digit zip code, some untaxed cities (e.g. Bainbridge Island) overlap with other untaxed 3-digit zip codes (e.g. Bainbridge Island includes areas in the 980 and 983 zip codes). Therefore, population estimates for untaxed cities in the "981" 3-digit zip code may include people from untaxed 3-digit zip codes. Because of this, the estimate of the % of the population covered by an SSB tax in the "981" 3-digit zip code is conservative (underestimated).

© 2024 Kaplan S et al. *JAMA Health Forum.*

**eTable 3.** Two-Way Fixed Effects Estimation Results for Composite and Individual City Analyses

| | Volume Purchases (Oz.) | Avg. Price per Oz. | Border Volume Purchases (Oz.) |
|---|---|---|---|
| **Composite (Balanced)** | -10,087,267.0*** | 0.0123*** | -2,486,372.0** |
| | (2,711,915.0) | (0.0026) | (883,365.0) |
| Dep. Var. Pretreatment Mean | 27,850,700 | 0.041 | 42,345,118 |
| Percent Change (%) | -36.22 | 30.38 | -5.87 |
| Observations | 16,980 | 16,980 | 21,984 |
| **Boulder** | -4,509,647.0*** | 0.0215*** | 906,480.0 |
| | (507,729.0) | (0.0003) | (1,000,519.0) |
| Dep. Var. Pretreatment Mean | 6,112,734 | 0.045 | 3,378,103 |
| Percent Change (%) | -73.77 | 47.39 | 26.83 |
| Observations | 27,440 | 27,440 | 36,162 |
| **San Francisco** | -12,068,979.0*** | 0.0142*** | -5,667,943.0** |
| | (586,217.0) | (0.0003) | (1,929,936.0) |
| Dep. Var. Pretreatment Mean | 31,0762,01 | 0.041 | 72,366,839 |
| Percent Change (%) | -38.84 | 34.33 | -7.83 |
| Observations | 27,440 | 27,440 | 36,162 |
| **Philadelphia** | -24,102,200.0*** | 0.0215*** | -4,808,432.0 |
| | (453,161.0) | (0.0003) | (2,464,901.0) |
| Dep. Var. Pretreatment Mean | 44,5485,78 | 0.032 | 42,233,523 |
| Percent Change (%) | -54.1 | 66.1 | -11.39 |
| Observations | 27,440 | 27,440 | 36,162 |
| **Oakland** | -4,114,207.0*** | 0.0092*** | -5,022,741.0 |
| | (507,729.0) | (0.0003) | (2,668,559.0) |
| Dep. Var. Pretreatment Mean | 15,561,263 | 0.038 | 16,544,381 |
| Percent Change (%) | -26.44 | 23.87 | -30.36 |
| Observations | 27,440 | 27,440 | 36,162 |
| **Seattle** | -12,428,611.0*** | 0.0057*** | -1,366,042.0 |
| | (586,217.0) | (0.0003) | (1,543,743.0) |
| Dep. Var. Pretreatment Mean | 41,954,725 | 0.045 | 38,603,038 |
| Percent Change (%) | -29.62 | 12.59 | -3.54 |
| Observations | 27,440 | 27,440 | 36,162 |

*Note:* $^{*}p<0.05$ $^{**}p<0.01$ $^{***}p<0.001$

All specifications include 3-digit zip code and month-year fixed effects. Standard errors are robustly estimated and clustered at the 3-digit zip code level.

© 2024 Kaplan S et al. *JAMA Health Forum.*

**eFigure 1.** Comparing Treated and Synthetic Values of Prognostic Factors from the Analysis of SSB Shelf Prices

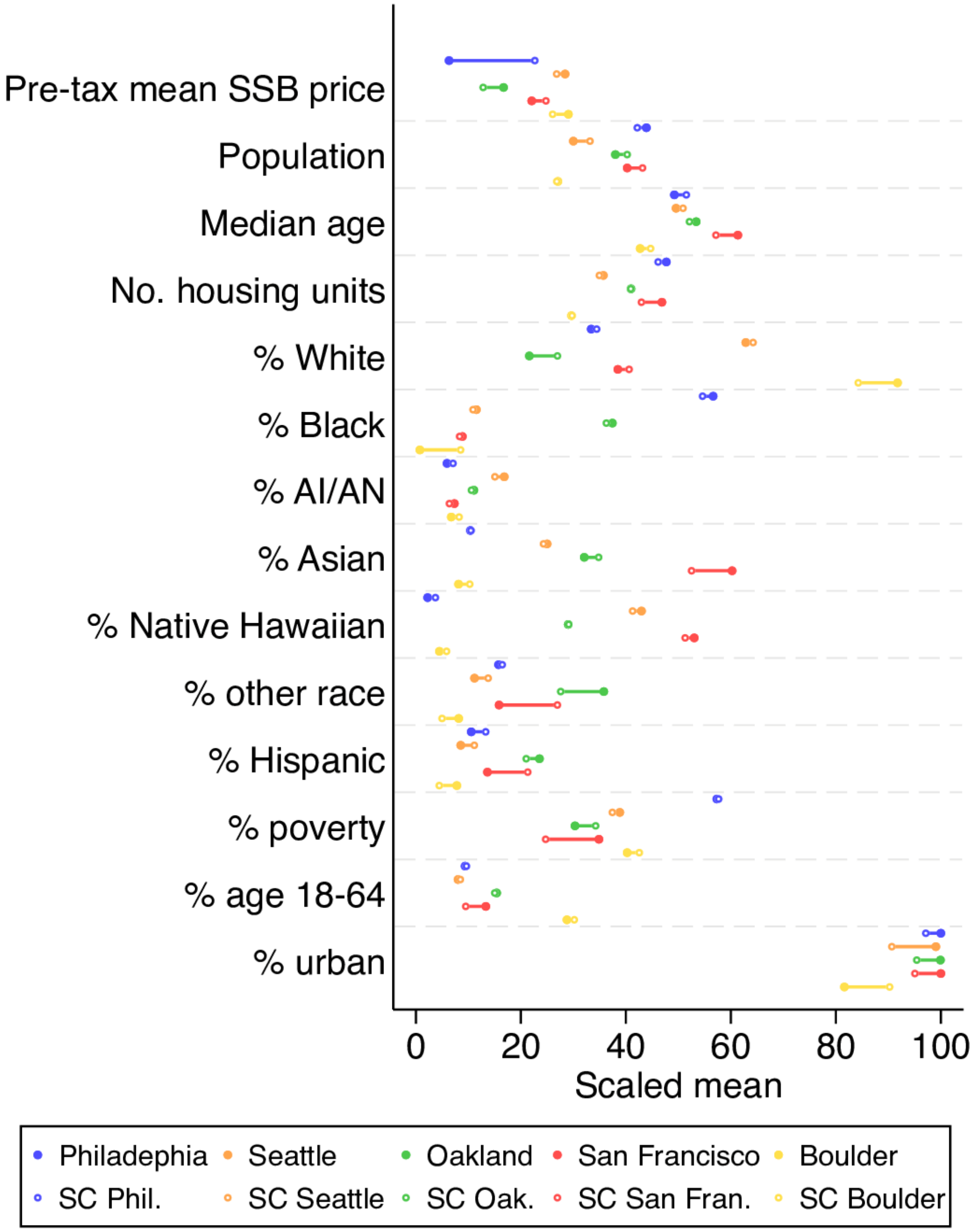


© 2024 Kaplan S et al. *JAMA Health Forum.*

**eFigure 2.** Overlap of US Census Sociodemographic Characteristics between each taxed city and the donor pool of control 3-digit zip codes

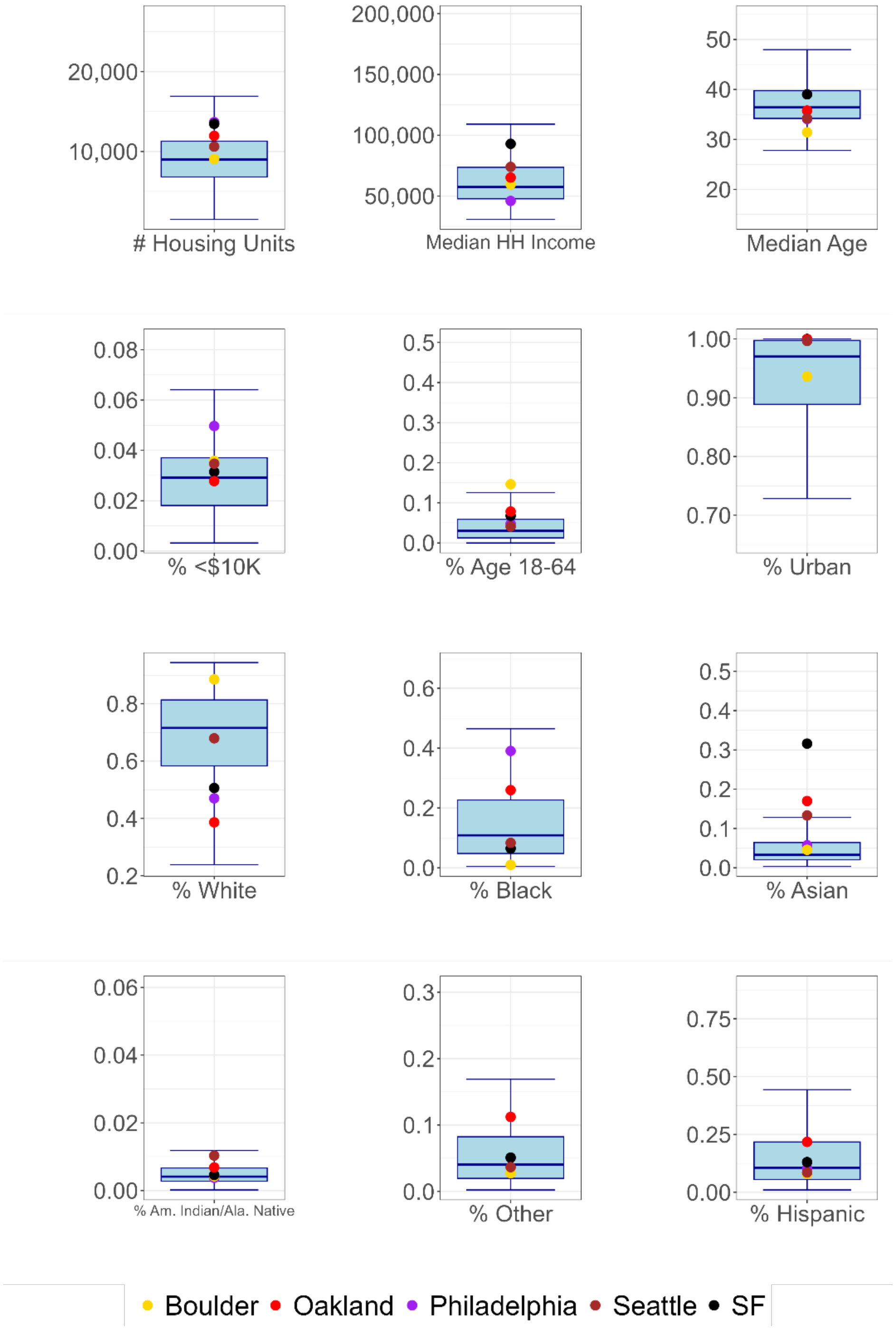


*Note*: Metrics for each 3-digit zip code were taken from either the 2010 Census or 2016 American Community Survey (ACS). Colored points on each plot represent values for each of the five treated localities. Box plots for each characteristic are formed from the distribution within the subsample of 3-digit zip codes used in the primary analysis.

© 2024 Kaplan S et al. *JAMA Health Forum.*

**eFigure 3.** Composite and Individual Locality Price Pass-Through

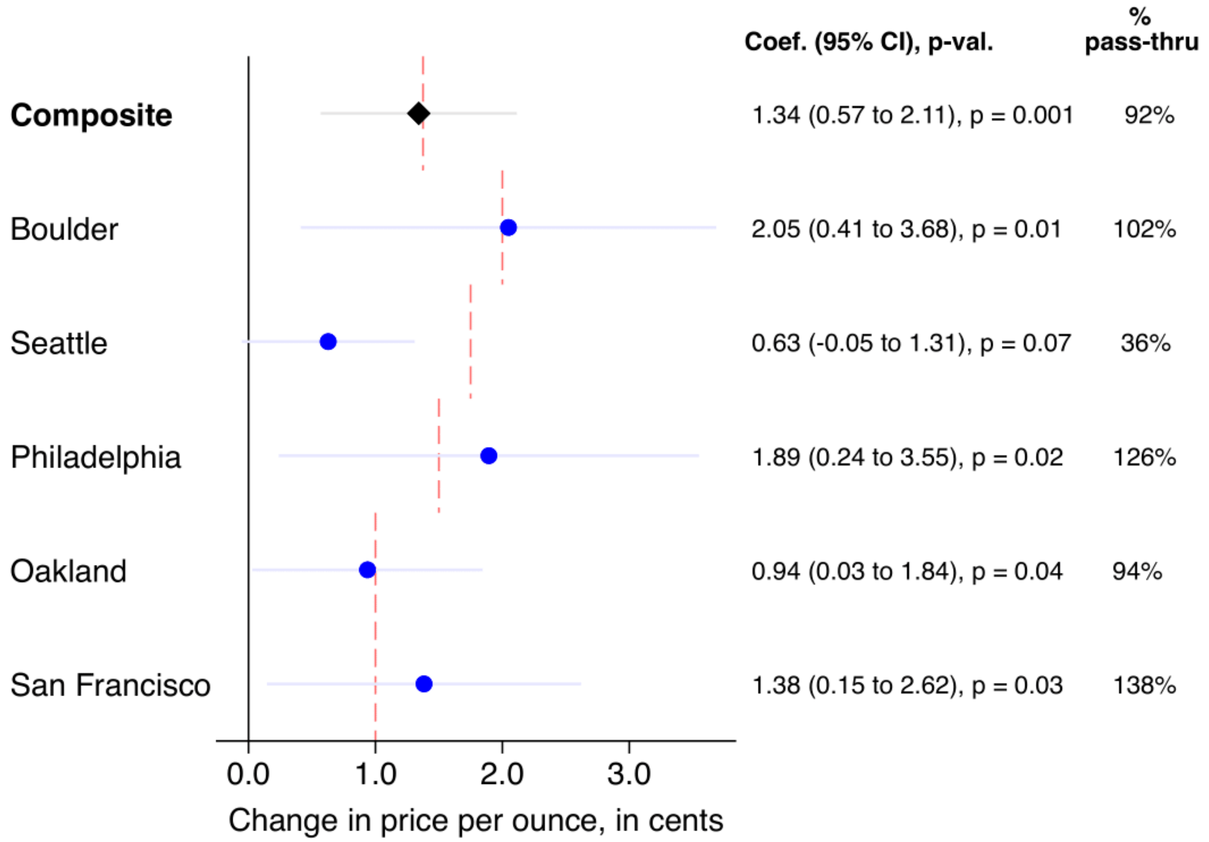


*Note*: Coefficient estimates represent the average total number of cents per ounce passed through to shelf prices of SSB products in the composite estimation and each individual treated locality. Dotted red lines denote full (100%) pass-through. Lightly shaded horizontal lines through each coefficient indicate 95% confidence intervals. % pass-thru indicates the % of the per-ounce tax in the composite estimation and each individual treated locality that was reflected in changes in shelf prices.

© 2024 Kaplan S et al. *JAMA Health Forum.*

**eFigure 4.** Composite and Individual Changes in Volume Sales in Adjacent Border Zip Codes

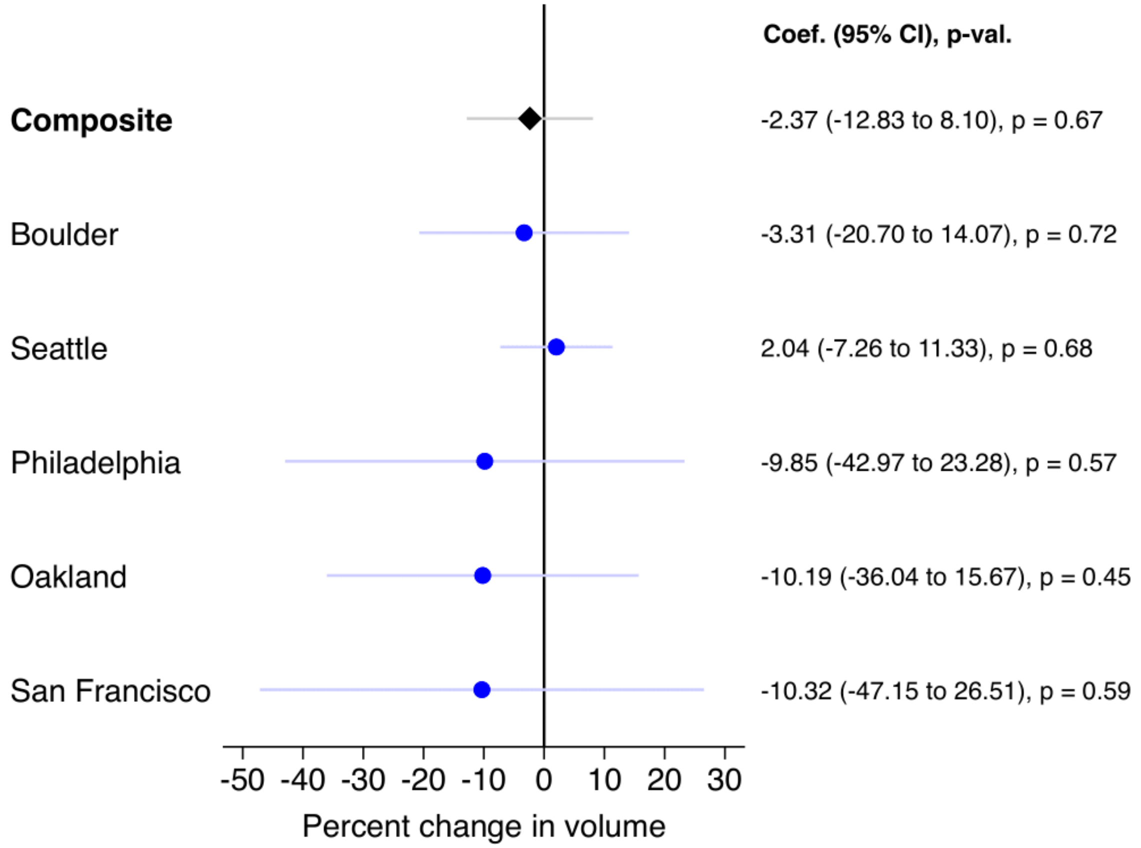


*Note*: Coefficient estimates represent the % change in SSB purchases in immediately adjacent border localities to each treated locality, and all borders in the composite estimation. Lightly shaded horizontal lines through each coefficient indicate 95% confidence intervals. Corresponding 95% confidence intervals and p-values are indicated next to each coefficient.

© 2024 Kaplan S et al. *JAMA Health Forum.*

**eFigure 5.** Augmented Synthetic Control Estimates for Individual Locality Changes in Price

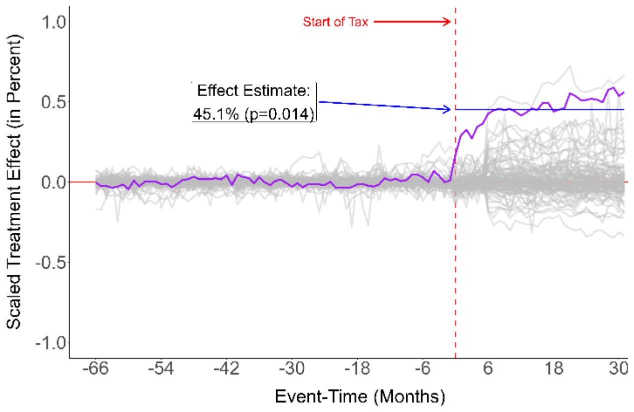


(a) 803 (Boulder)

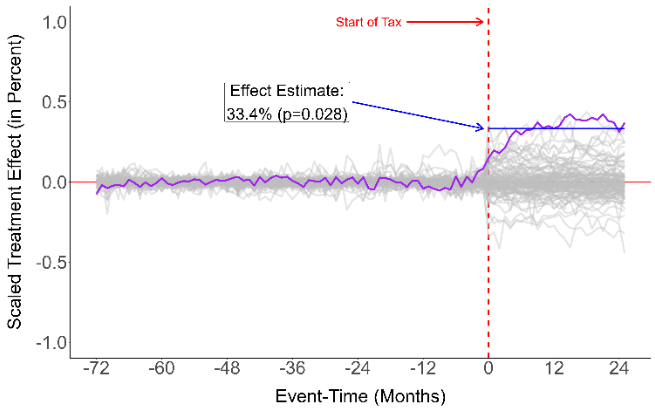


(b) 941 (San Francisco)

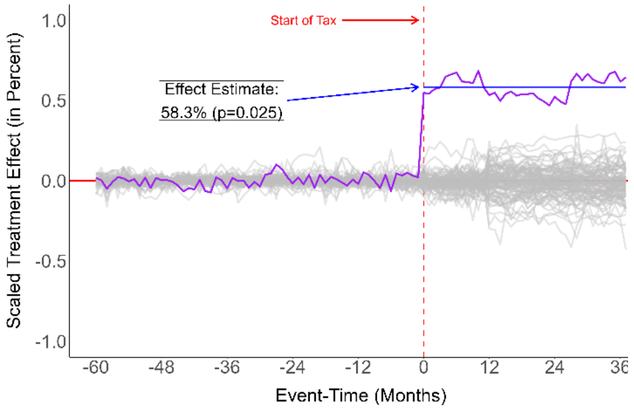


(c) 191 (Philadelphia)

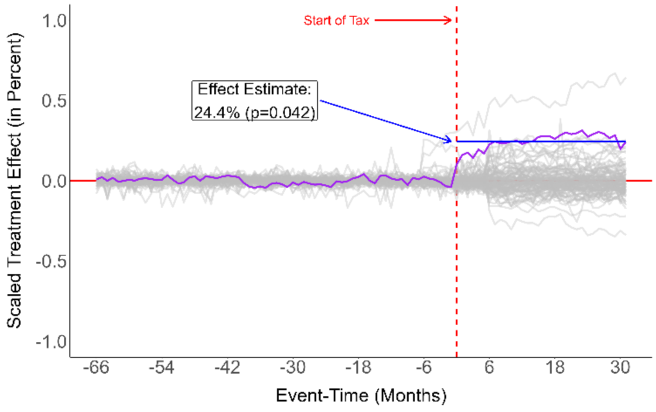


(d) 946 (Oakland)

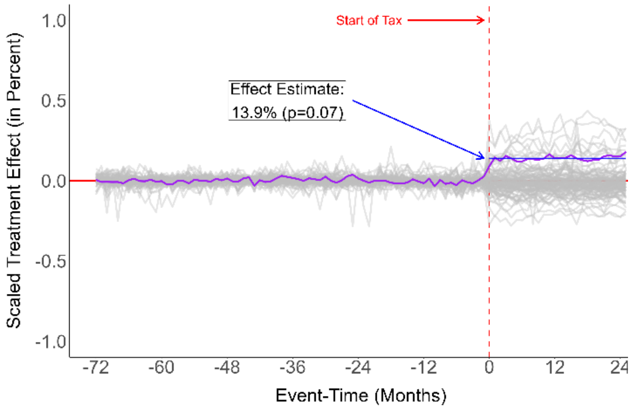


(e) 981 (Seattle)

© 2024 Kaplan S et al. *JAMA Health Forum.*

**eFigure 6.** Augmented Synthetic Control Estimates for Individual Locality Changes in Volume Sales

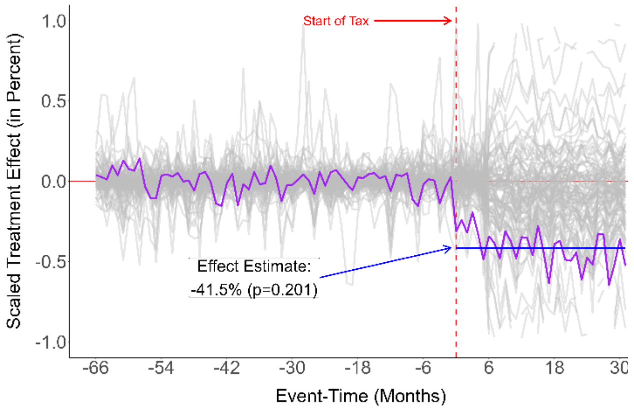


(a) 803 (Boulder)

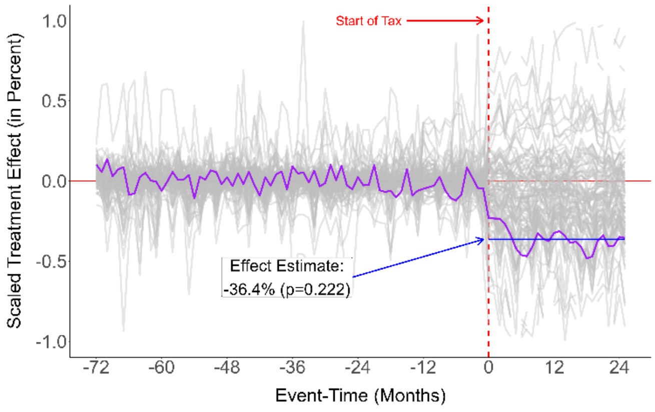


(b) 941 (San Francisco)

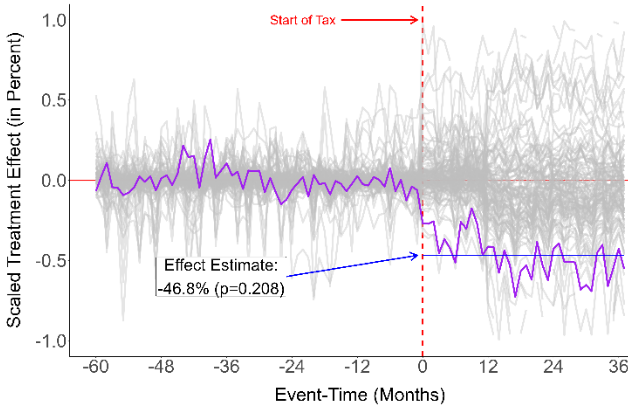


(c) 191 (Philadelphia)

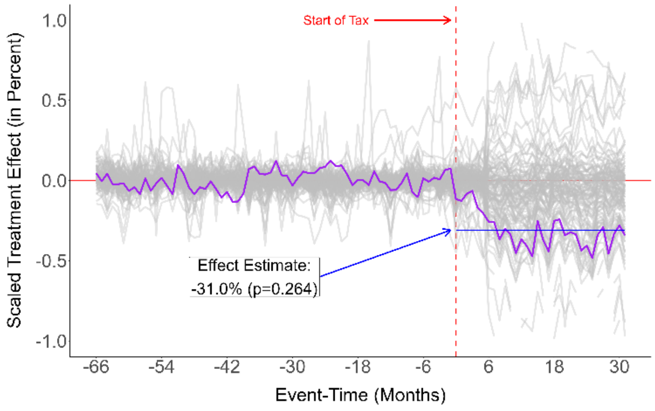


(d) 946 (Oakland)

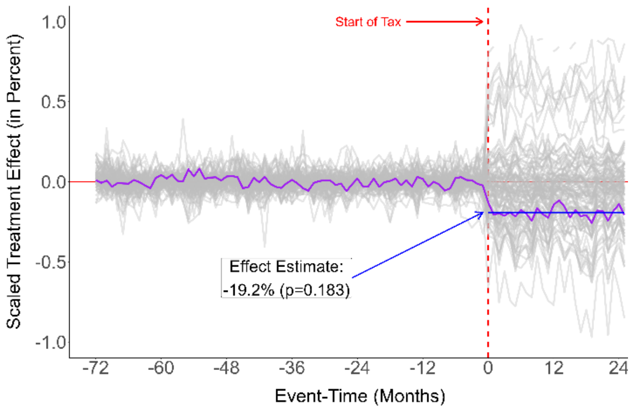


(e) 981 (Seattle)

© 2024 Kaplan S et al. *JAMA Health Forum.*

**eFigure 7.** Augmented Synthetic Control Estimates of Individual Locality Changes in Volume Sales of SSB Products in Border Areas

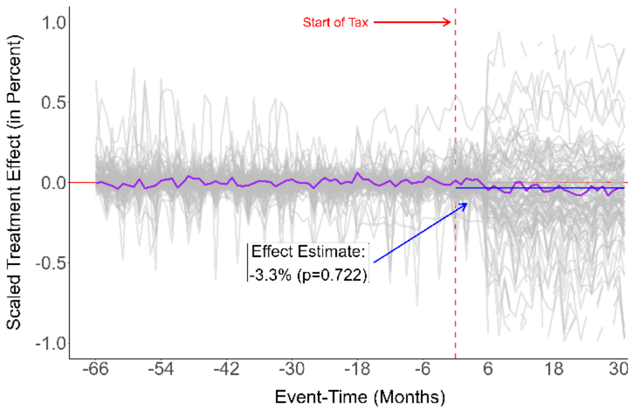


(a) 803 (Boulder)

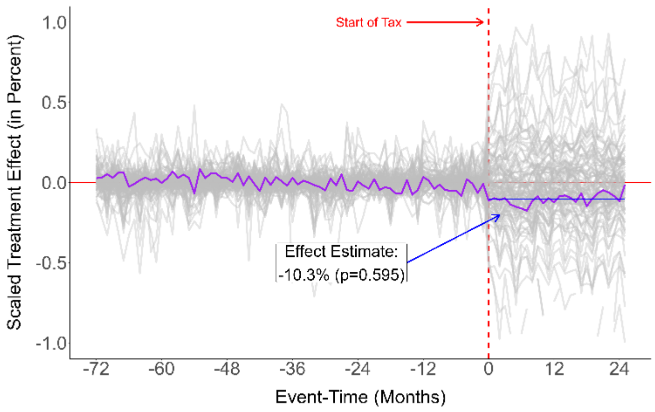


(b) 941 (San Francisco)

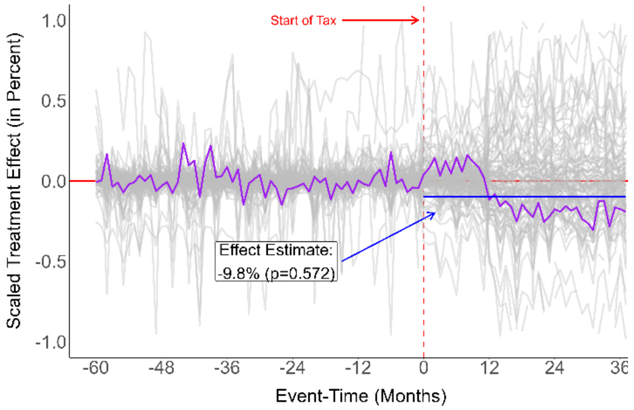


(c) 191 (Philadelphia)

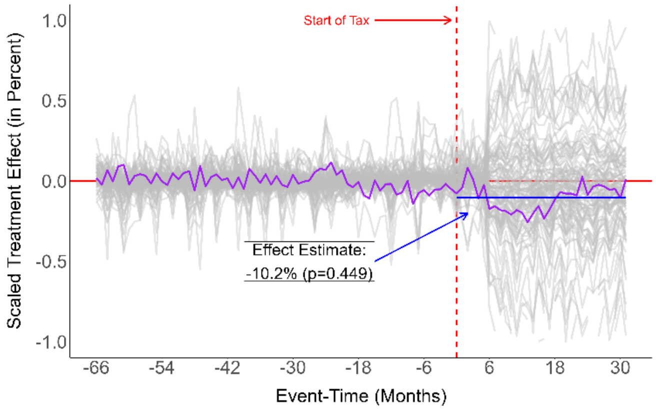


(d) 946 (Oakland)

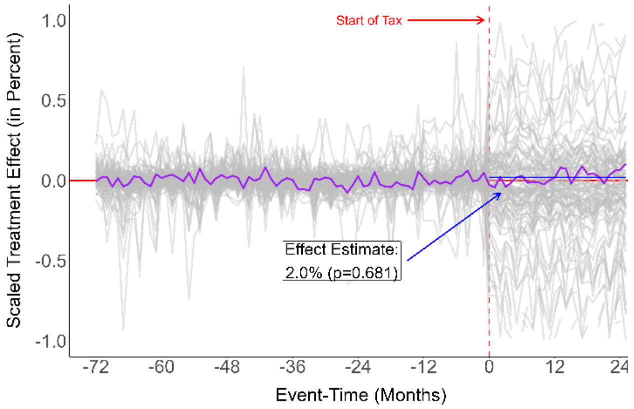


(e) 981 (Seattle)

© 2024 Kaplan S et al. *JAMA Health Forum.*

**eFigure 8.** Augmented Synthetic Control Estimates for Composite Changes in Price and Volume Sales of SSB Products (Population Weighted)

Panel A. Changes in SSB Prices

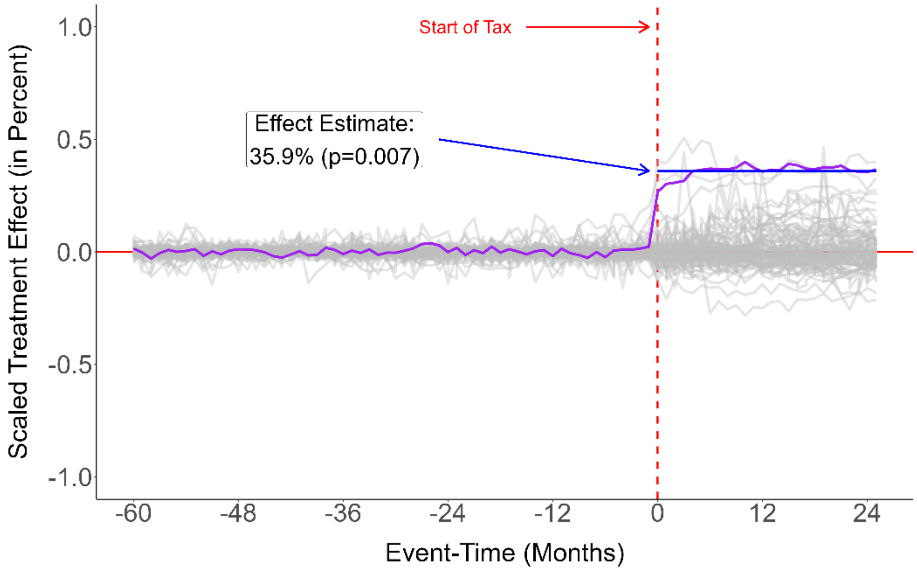


Panel B. Changes in SSB Volume Sales

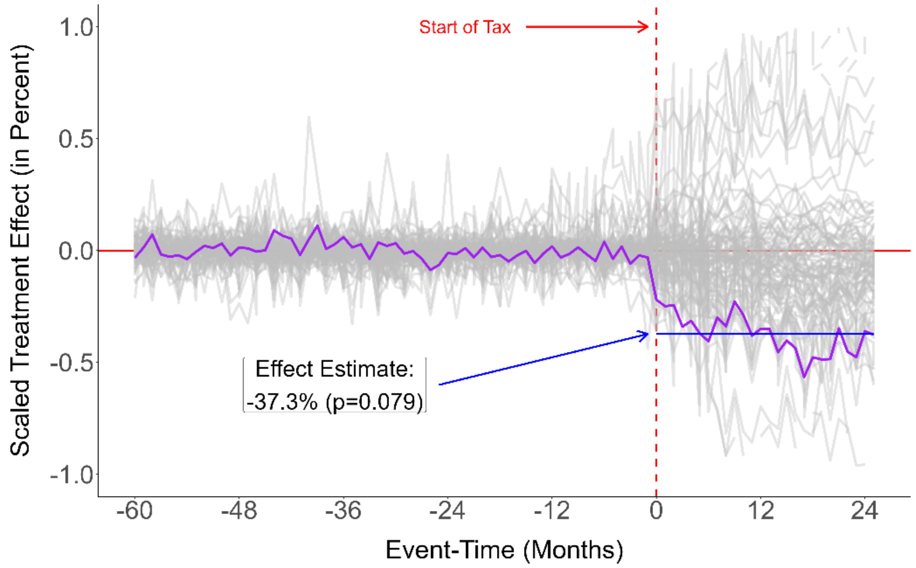


*Note*: Panel a) shows the % change in volume sold (in ounces), and panel b) the % change in shelf prices in response to implementing an excise SSB tax for the staggered adoption composite analysis. The bolded purple line represents the composite treated unit, while the lightly shaded grey lines represent in-space placebo estimates from the donor pool. The composite effect is explicitly weighted by the population of each individual treated city. % changes are calculated with respect to the population-weighted average of the pre-treatment means of each of the five treated localities. The composite effect size estimates and p-values are provided in the designated box of each panel.

© 2024 Kaplan S et al. *JAMA Health Forum.*

**eFigure 9.** Augmented Synthetic Control Estimates of Composite Changes in Volume Sales of SSB Products in Border Areas (Population Weighted)

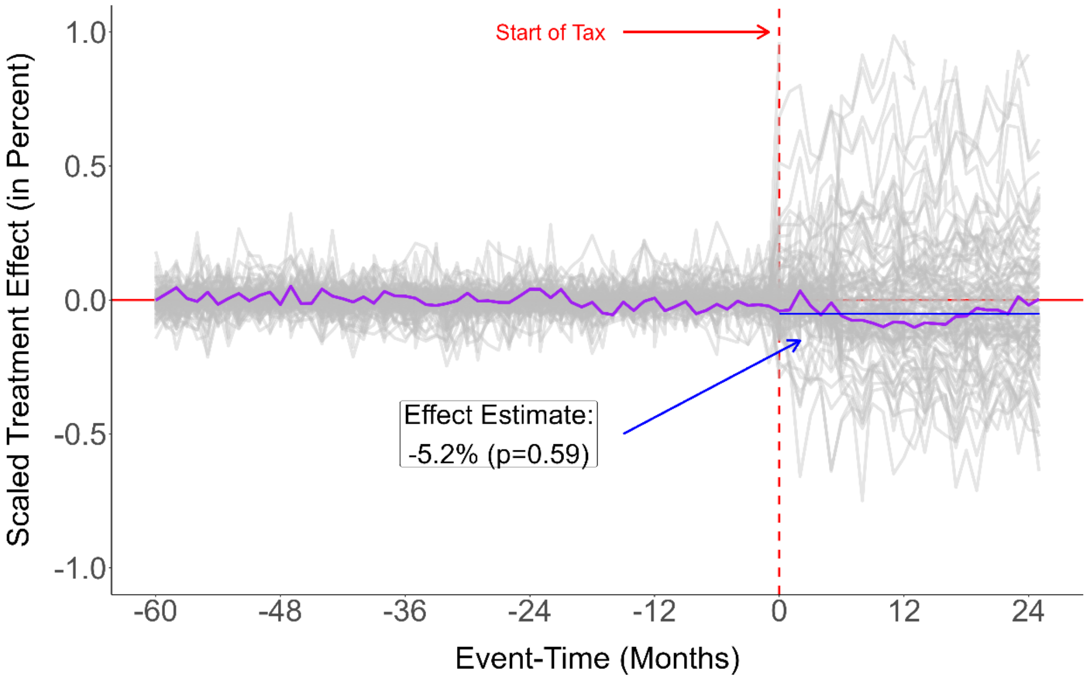


*Note*: This figure shows the staggered adoption composite analysis % change in volume sold (in ounces) in immediately adjacent bordering 3-digit zip codes in response to implementing an excise SSB tax in the five treated zip codes. The bolded purple line represents the composite adjacent border unit, while the lightly shaded grey lines represent in-space placebo estimates from the donor pool. The composite effect is explicitly weighted by the population of each individual treated city. % changes are calculated with respect to the population-weighted average of the pre-treatment means of each of the twelve adjacent border localities. The composite effect size estimates and p-values are provided in the designated box.

© 2024 Kaplan S et al. *JAMA Health Forum.*

**eFigure 10.** Composite and Individual Locality Demand Elasticity Estimates (Urbanicity > 0.85)

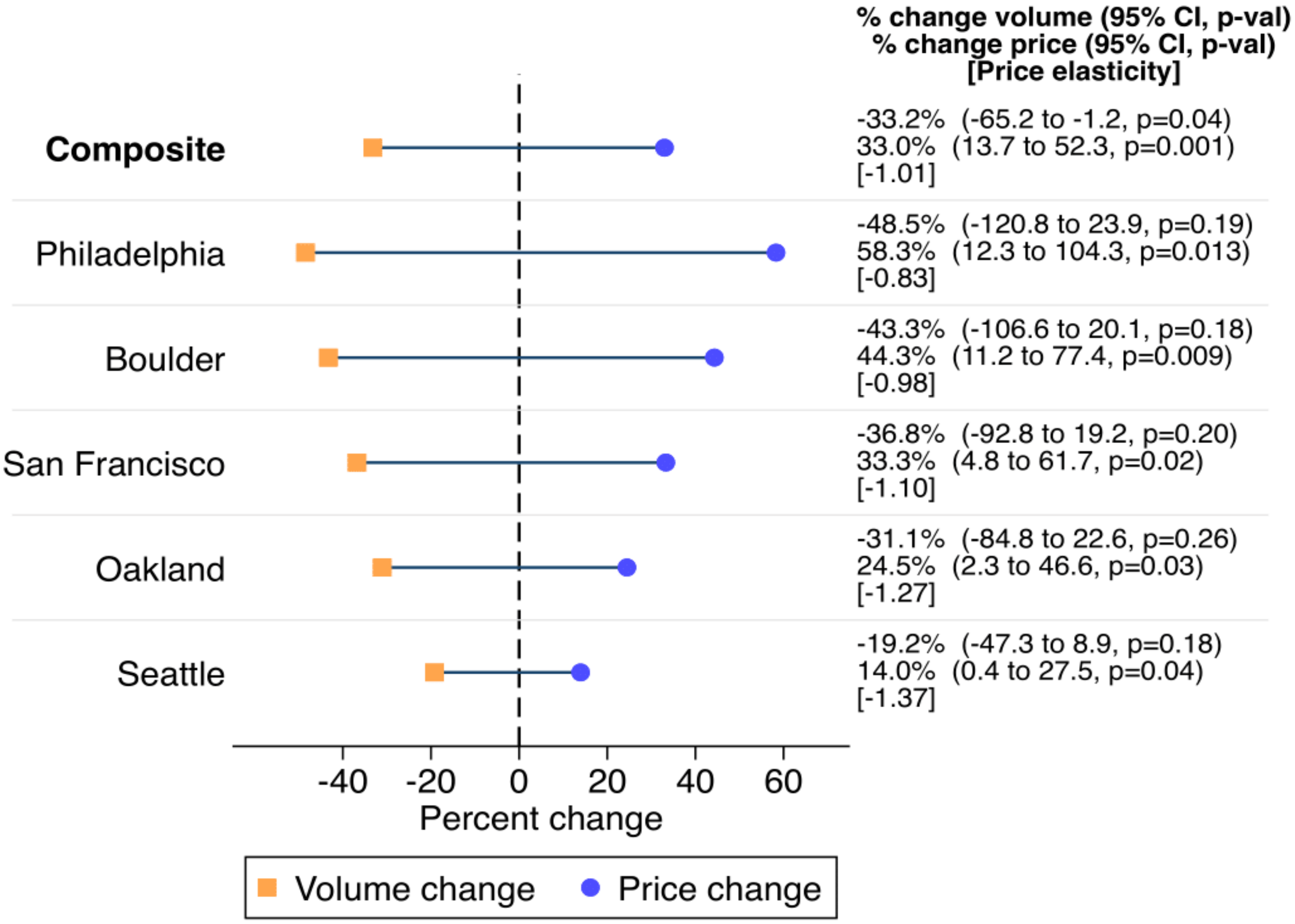


*Note*: This plot shows the % change in volume sold (in ounces) and % change in price for the augmented synthetic control composite analysis, and the same information for augmented synthetic control analyses of each of the five treated localities individually. Price elasticities of demand are provided in brackets, and 95% confidence intervals and p-values for each estimation are provided in parentheses.

© 2024 Kaplan S et al. *JAMA Health Forum.*

**eFigure 11.** Augmented Synthetic Control Estimates for Composite Changes in Price and Volume Sales of SSB Products (Urbanicity > 0.85)

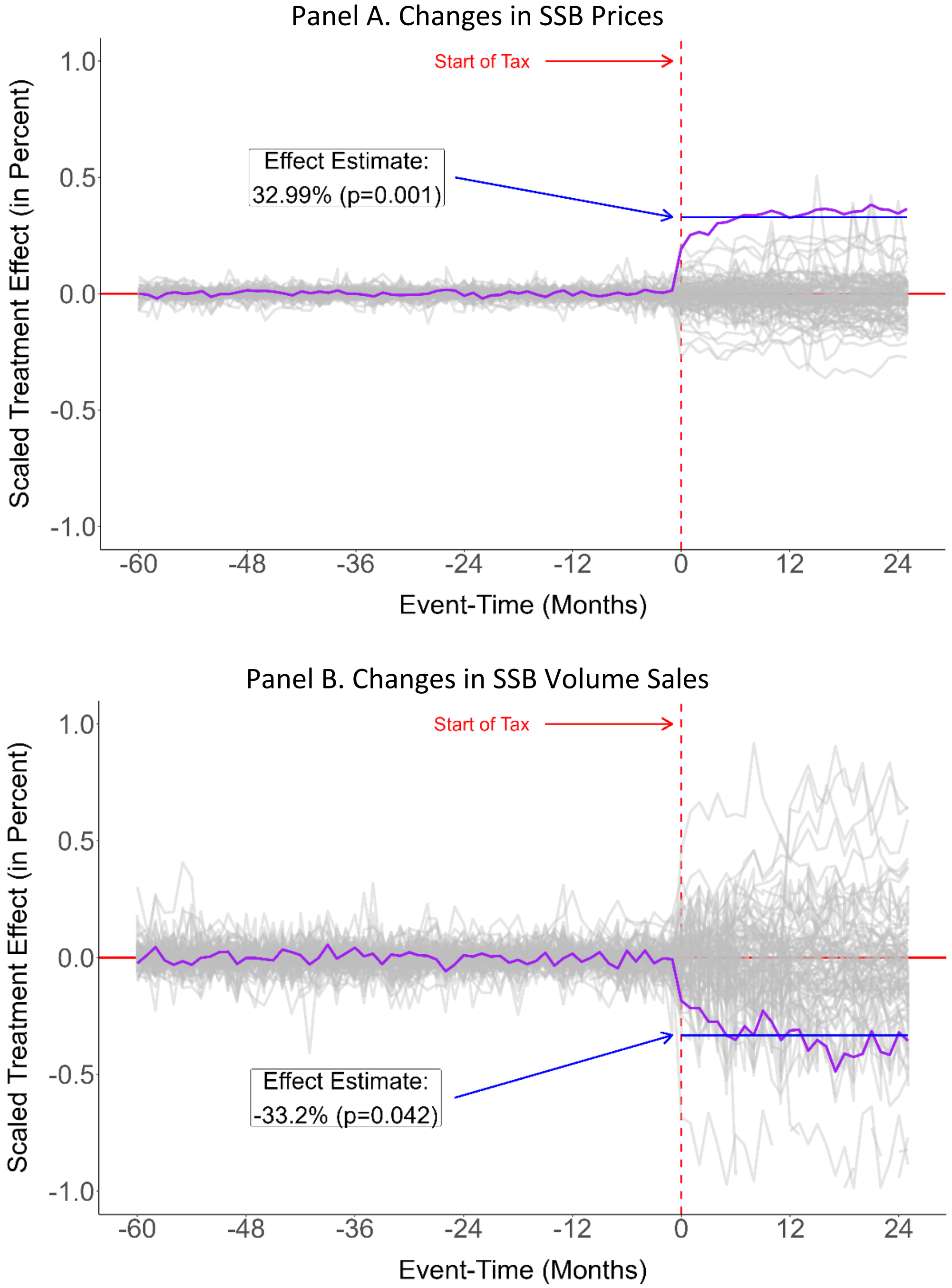


*Note*: Panel a) shows the % change in price and panel b) the % change in volume in response to the implementation of an excise SSB tax for the composite analysis. The bolded purple line represents the composite treated unit, while the lightly shaded grey lines represent in-space placebo estimates from the donor pool. % changes are calculated with respect to the average of the pre-treatment means of each of the five treated localities. The average composite effect estimates and p-values are provided in the designated box of each panel.

© 2024 Kaplan S et al. *JAMA Health Forum.*

**eFigure 12.** Augmented Synthetic Control Estimates of Composite Changes in Volume Sales of SSB Products in Border Areas (Urbanicity > 0.85)

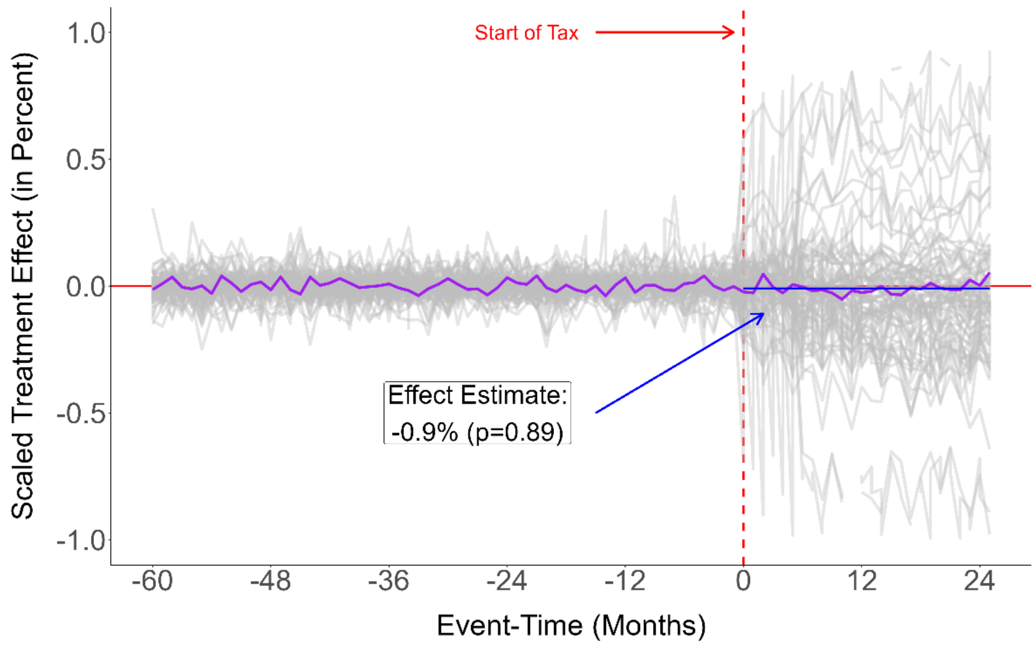


*Note*: This figure shows the composite analysis % change in volume sold in immediately adjacent bordering 3-digit zip codes in response to the implementation of an excise SSB tax in the five treated zip codes. The bolded purple line represents the composite adjacent border unit, while the lightly shaded grey lines represent in-space placebo estimates from the donor pool. % changes are calculated with respect to the average of the pre-treatment means of each of the twelve adjacent border localities. The average composite effect estimates and p-values are provided in the designated box.

© 2024 Kaplan S et al. *JAMA Health Forum.*

**eFigure 13.** Composite and Individual Locality Demand Elasticity Estimates (Urbanicity > 0.9)

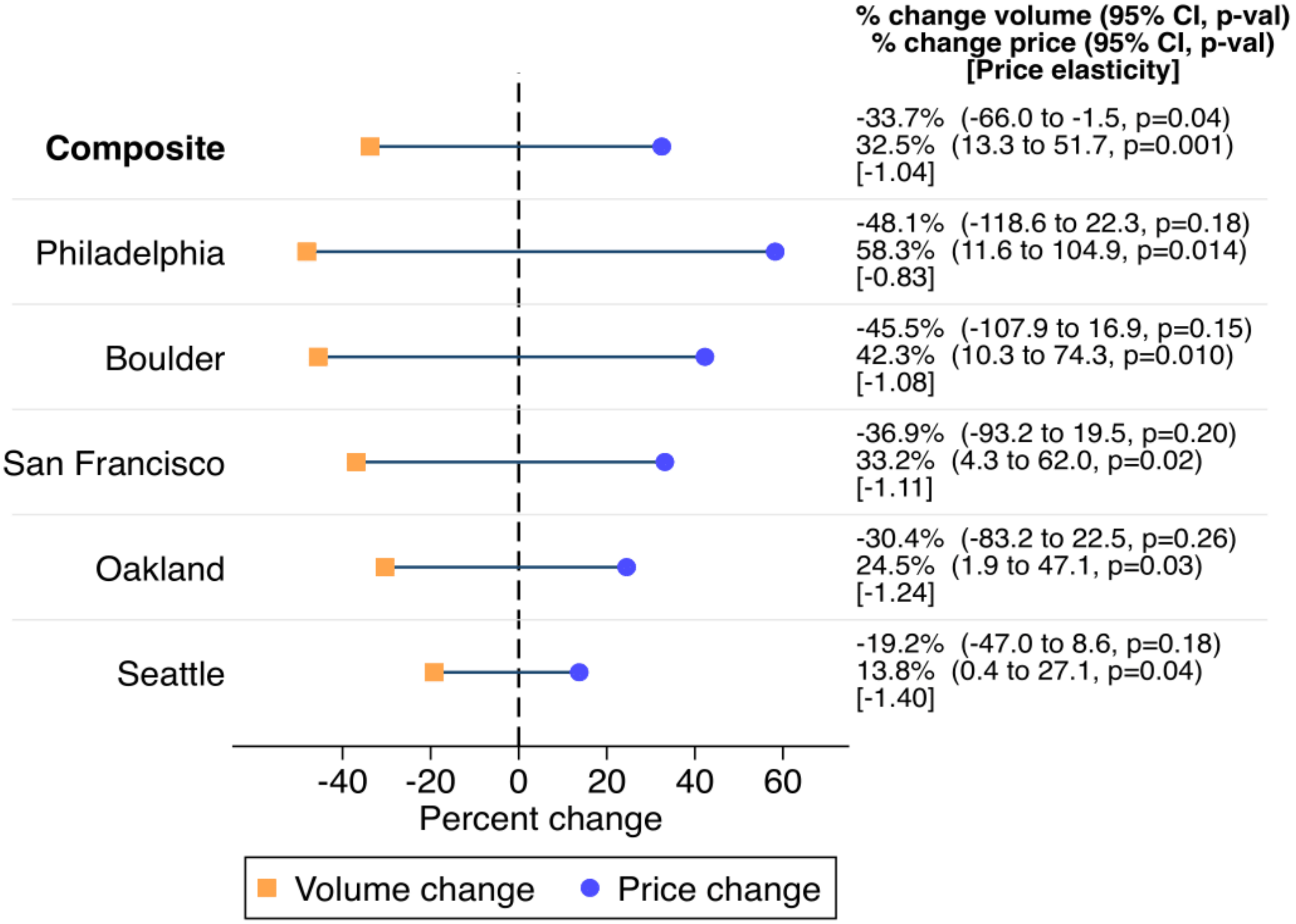


*Note*: This plot shows the % change in volume sold (in ounces) and % change in price for the augmented synthetic control staggered adoption composite analysis, and the same information for augmented synthetic control analyses of each of the five treated localities individually. Price elasticities of demand are provided in brackets, and 95% confidence intervals and p-values for each estimation are provided in parentheses.

© 2024 Kaplan S et al. *JAMA Health Forum.*

**eFigure 14.** Augmented Synthetic Control Estimates for Composite Changes in Price and Volume Sales of SSB Products (Urbanicity > 0.9)

Panel A. Changes in SSB Prices

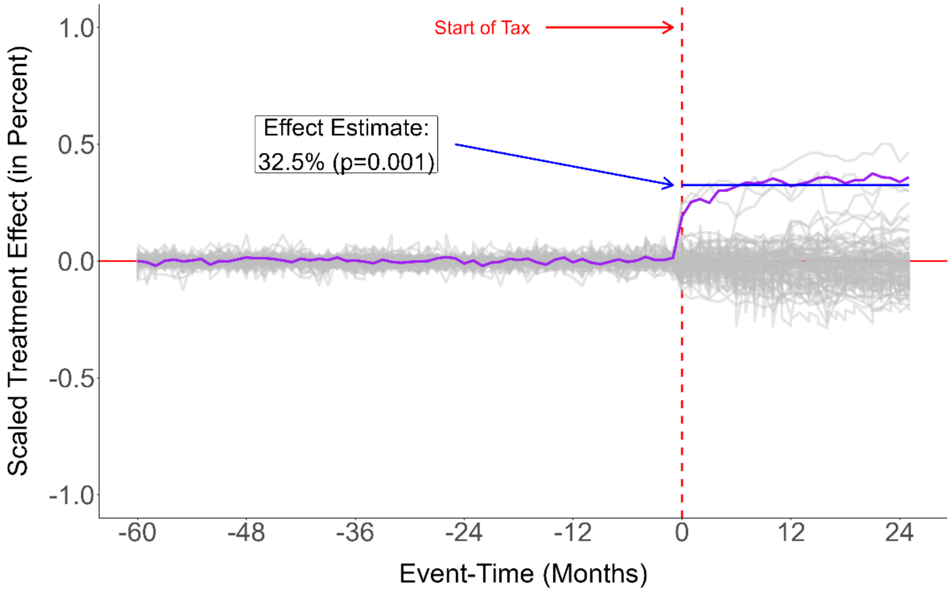


Panel B. Changes in SSB Volume Sales

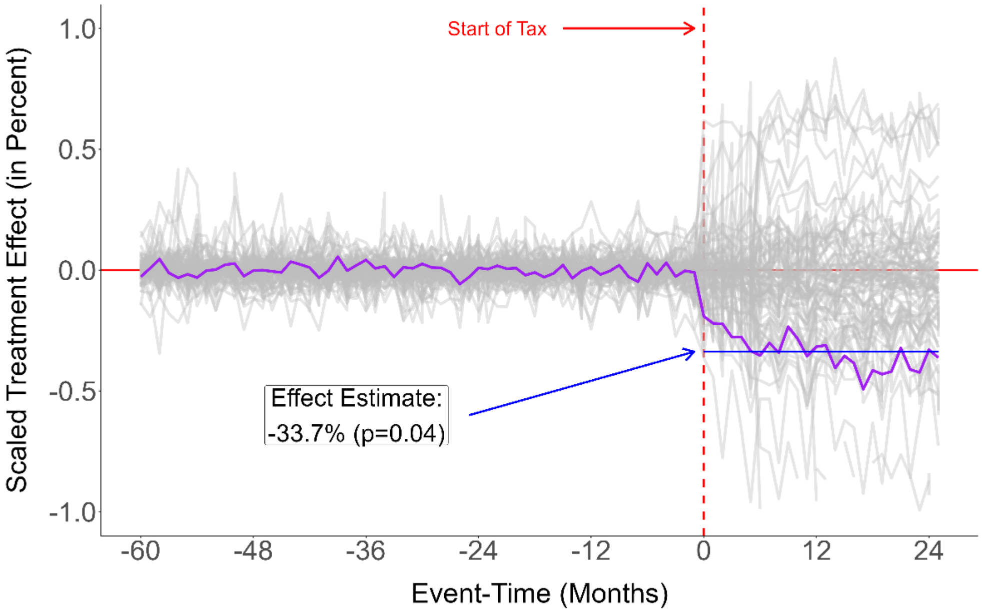


*Note*: Panel a) shows the % change in price and panel b) the % change in volume sold (in ounces) in response to the implementation of an excise SSB tax for the composite analysis. The bolded purple line represents the composite treated unit, while the lightly shaded grey lines represent in-space placebo estimates from the donor pool. % changes are calculated with respect to the average of the pre-treatment means of each of the five treated localities. The average composite effect estimates and p-values are provided in the designated box of each panel.

© 2024 Kaplan S et al. *JAMA Health Forum.*

**eFigure 15.** Augmented Synthetic Control Estimates of Composite Changes in Volume Sales of SSB Products in Border Areas (Urbanicity > 0.9)

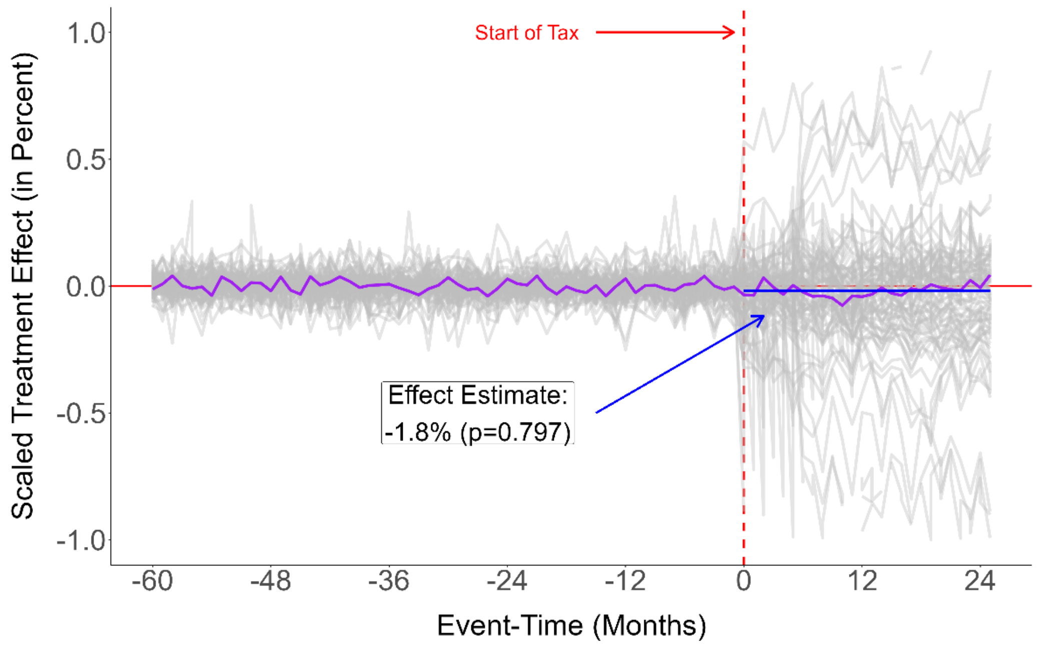


*Note*: This figure shows the composite analysis % change in volume sold in immediately adjacent bordering 3-digit zip codes in response to the implementation of an excise SSB tax in the five treated zip codes. The bolded purple line represents the composite adjacent border unit, while the lightly shaded grey lines represent in-space placebo estimates from the donor pool. % changes are calculated with respect to the average of the pre-treatment means of each of the twelve adjacent border localities. The average composite effect estimates and p-values are provided in the designated box.

© 2024 Kaplan S et al. *JAMA Health Forum.*

**eFigure 16.** TWFE Estimates of Composite Changes in Prices, Volume Sales, and Border Volume Sales

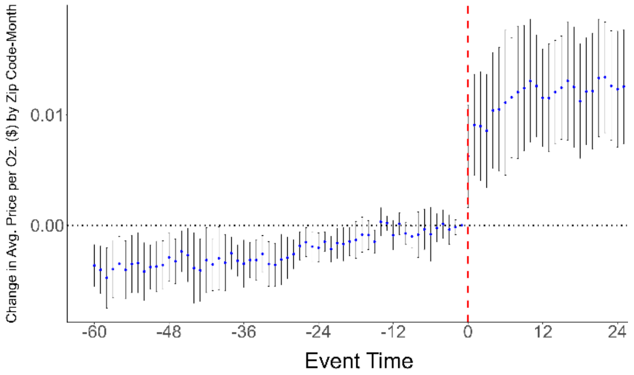


Panel A. Changes in SSB Prices

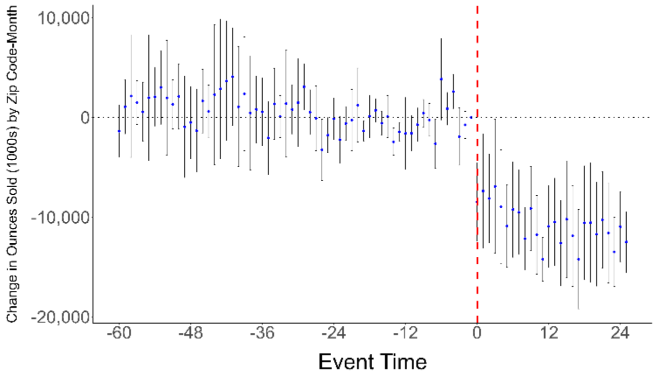


Panel B. Changes in SSB Volume Sales

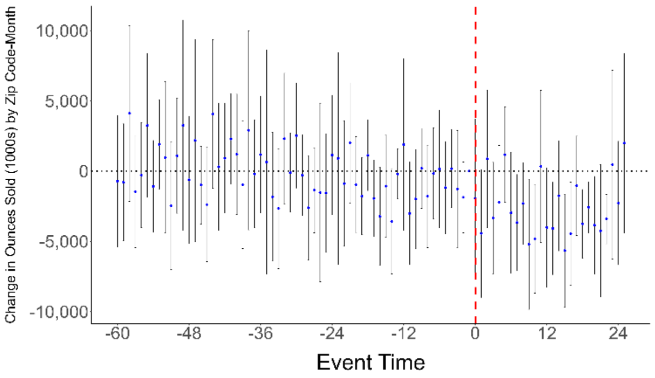


Panel C. Changes in Border SSB Volume Sales

*Note*: All point estimates should be interpreted relative to the omitted event-time period (-1). 95% CIs are depicted with each point estimate. The red dashed line indicates timing of policy enactment.

© 2024 Kaplan S et al. *JAMA Health Forum.*

**eFigure 17.** TWFE Estimates of Individual Locality Changes in Prices

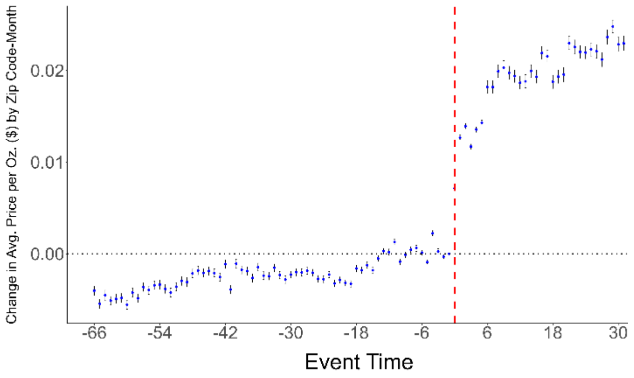


(a) 803 (Boulder)

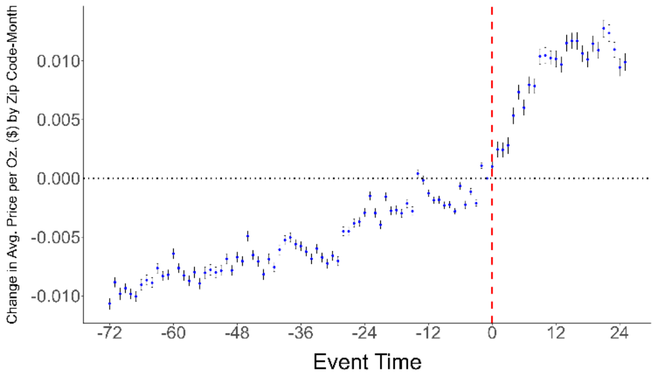


(b) 941 (San Francisco)

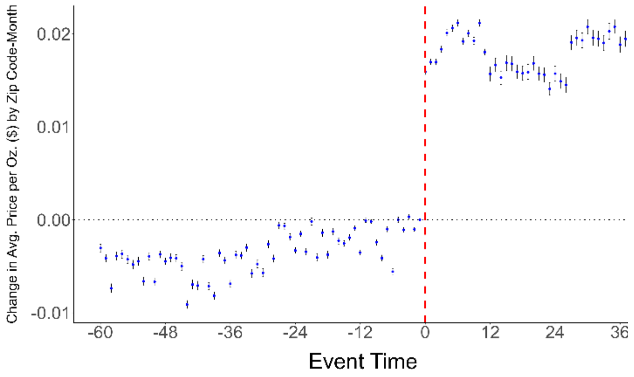


(c) 191 (Philadelphia)

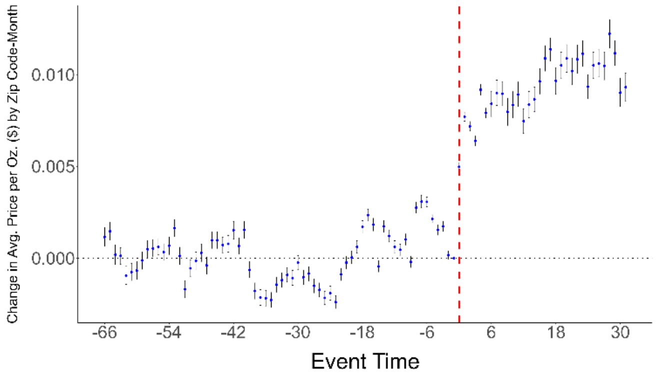


(d) 946 (Oakland)

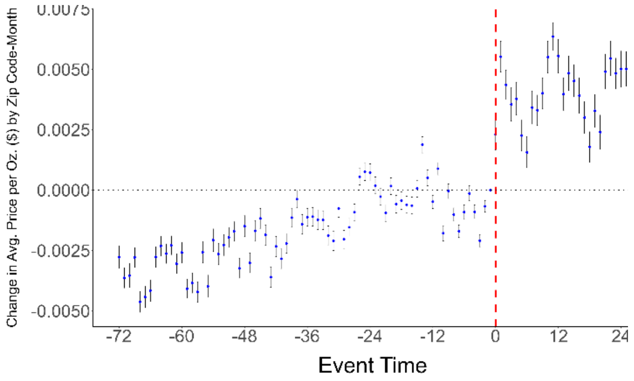


(e) 981 (Seattle)

*Note*: All point estimates should be interpreted relative to the omitted event-time period (-1). 95% CIs are depicted with each point estimate. The red dashed line indicates timing of policy enactment.

© 2024 Kaplan S et al. *JAMA Health Forum.*

**eFigure 18.** TWFE Estimates of Individual Locality Changes in Volume Sales

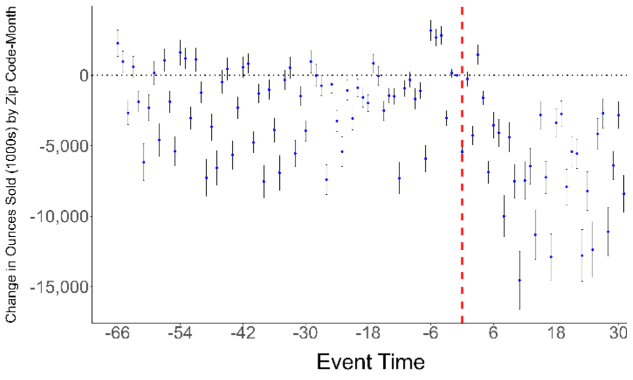


(a) 803 (Boulder)

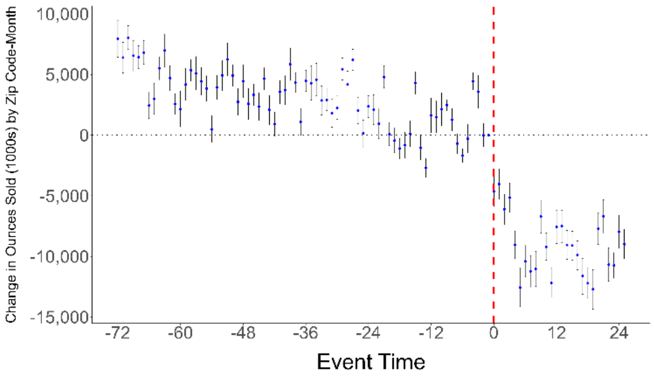


(b) 941 (San Francisco)

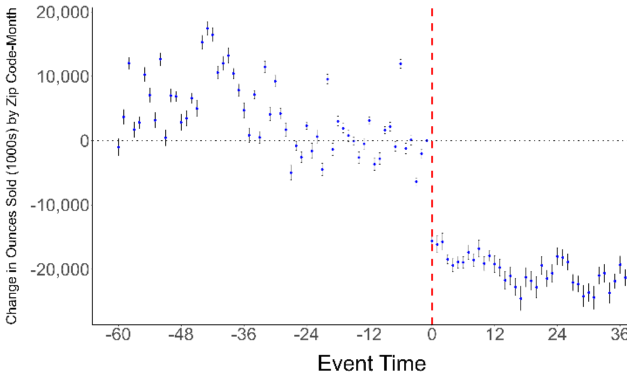


(c) 191 (Philadelphia)

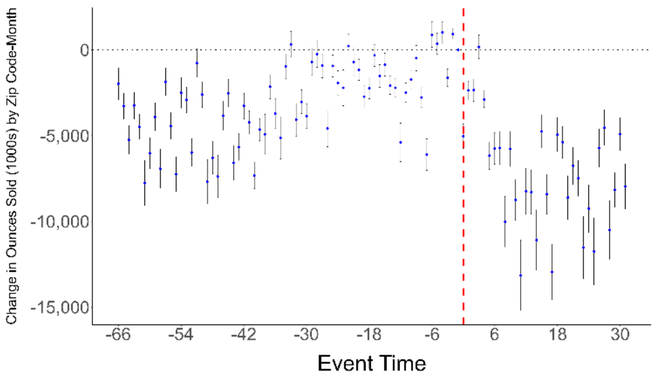


(d) 946 (Oakland)

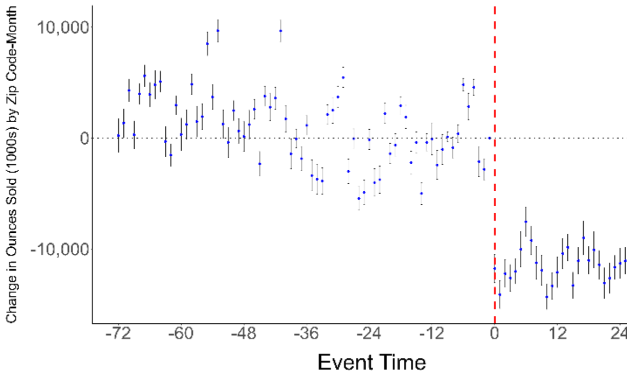


(e) 981 (Seattle)

*Note*: All point estimates should be interpreted relative to the omitted event-time period (-1). 95% CIs are depicted with each point estimate. The red dashed line indicates timing of policy enactment.

© 2024 Kaplan S et al. *JAMA Health Forum.*

**eFigure 19.** TWFE Estimates of Individual Locality Changes in Volume Sales of SSB Products in Border Areas

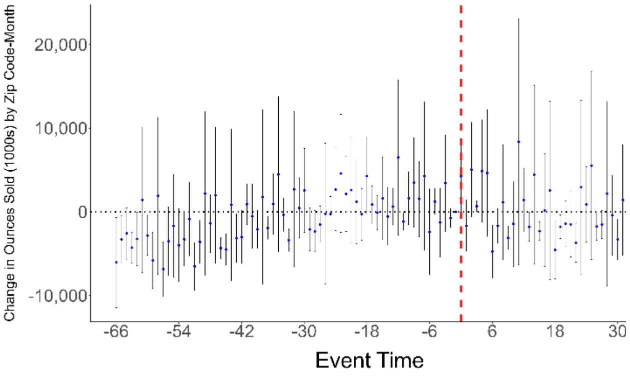


(a) 803 (Boulder)

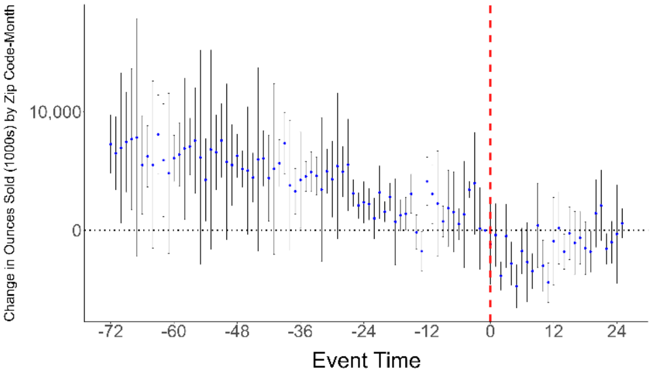


(b) 941 (San Francisco)

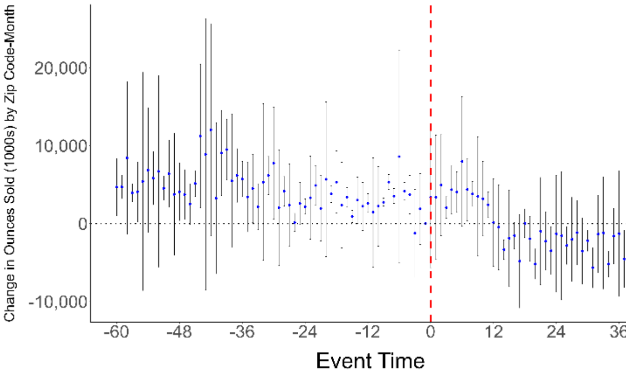


(c) 191 (Philadelphia)

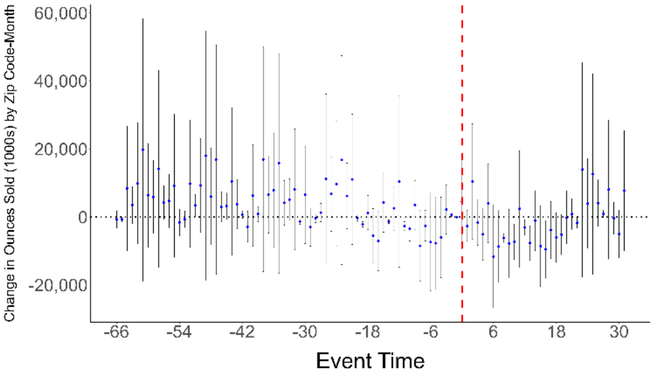


(d) 946 (Oakland)

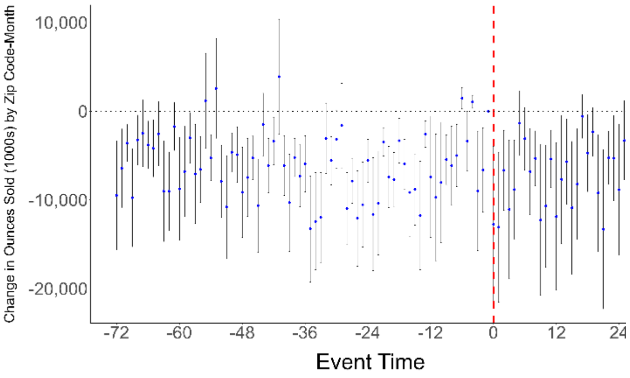


(e) 981 (Seattle)

*Note*: All point estimates should be interpreted relative to the omitted event-time period (-1). 95% CIs are depicted with each point estimate. The red dashed line indicates timing of policy enactment.

© 2024 Kaplan S et al. *JAMA Health Forum.*
