# Supplementary material for: Evaluation of Changes in Prices and Purchases Following Implementation of Sugar-Sweetened Beverage Taxes Across the US
Source: JAMA Health Forum. 2024 Jan 5;5(1):e234737. doi: 10.1001/jamahealthforum.2023.4737 (PMC10770775; doi:10.1001/jamahealthforum.2023.4737)
Supplement: Supplement 2. — Data Sharing Statement [file jamahealthforum-e234737-s002.pdf]

Data Sharing Statement

Data
Data available: No

Additional Information
Explanation for why data not available: Retail sales data from Nielsen are proprietary and subject to the terms of Nielsen's data use agreement with the Kilts Center at the University of Chicago. The data may be accessed by applying through the Kilts Center. Initial contact can be made using the email marketingdata@chicagobooth.edu or by visiting https://www.chicagobooth.edu/research/kilts/datasets/nielseniq-nielsen/subscribing.
